# Diagnostic accuracy of history taking, physical examination and imaging for non-chronic finger, hand and wrist ligament and tendon injuries: a systematic review update

**DOI:** 10.1136/bmjopen-2020-037810

**Published:** 2020-11-05

**Authors:** Patrick Krastman, Nina M C Mathijssen, Sita M A Bierma-Zeinstra, Gerald A Kraan, Jos Runhaar

**Affiliations:** 1 Department of General Practice, Erasmus MC University Medical Center, Rotterdam, The Netherlands; 2 Department of Orthopaedic, Reinier de Graaf Gasthuis, Delft, The Netherlands; 3 Department of Orthopedics, Erasmus MC University Medical Center, Rotterdam, The Netherlands

**Keywords:** diagnostic radiology, hand & wrist, adult orthopaedics

## Abstract

**Objective:**

The diagnostic work-up for ligament and tendon injuries of the finger, hand and wrist consists of history taking, physical examination and imaging if needed, but the supporting evidence is limited. The main purpose of this study was to systematically update the literature for studies on the diagnostic accuracy of tests for detecting non-chronic ligament and tendon injuries of the finger, hand and wrist.

**Methods:**

Medline, Embase, Cochrane Library, Web of Science, Google Scholar ProQuest and Cinahl were searched from 2000 up to 6 February 2019 for identifying studies. Methodological quality was assessed using the Quality Assessment of Diagnostic Accuracy Studies 2 checklist, and sensitivity (Se), specificity (Sp), accuracy, positive predictive value (PPV) and negative predictive value (NPV) were extracted.

**Results:**

None of the studies involved history taking. Physical examination, for diagnosing lesions of the triangular fibrocartilage complex (TFCC), showed Se, Sp, accuracy, PPV and NPV ranging from 58% to 90%, 20% to 69%, 56% to 73%, 53% to 71% and 55% to 65%, respectively. Physical examination in hand and finger injuries the Se, Sp, accuracy, PPV and NPV ranged from 88% to 99%, 75% to 100%, 34% to 88%, 91% to 100% and 75% to 95%, respectively. The accuracy of MRI with high-resolution (3 T) techniques for TFCC and interosseous ligaments of the proximal carpal row ranged from 89% to 91% and 75% to 100%, respectively. The accuracy of MRI with low-resolution (1.5 T) techniques for TFCC and interosseous ligaments of the proximal carpal row ranged from 81% to 100% and 67% to 95%, respectively.

**Conclusions:**

There is limited evidence on the diagnostic accuracy of history taking and physical examination for non-chronic finger, hand and wrist ligament and tendon injuries. Although some imaging modalities seemed to be acceptable for the diagnosis of ligament and tendon injuries in the wrist in patients presenting to secondary care, there is no evidence-based advise possible for the diagnosis of non-chronic finger, hand or wrist ligament and tendon injuries in primary care.

**Strengths and limitations of this study**

- This is the first study that systematically reviewed the accuracy of diagnostic tests for non-chronic hand and finger injuries, next to previously described accuracy of diagnostic tests for non-chronic wrist injuries.
- Studies on wrist injuries published before 2000 were not evaluated and not included in the current systematic review, as these were adequately described in published systematic reviews.
- Diagnostic tests heterogeneity precluded meta-analysis, caused by the fact that studies that evaluated the same pathologies showed marked diversity in population, index tests, reference test and methodological quality.

## Introduction

Wrist injuries are one of the most common presentations to the emergency department (ED) due to trauma and they commonly affect young people of working age.^1 2^ In the Netherlands, 21% of the patients initially consulted their general practitioner (GP) after a wrist injury, 41% went directly to an outpatient clinic and 35% had no further treatment.^3^ Within the GP’s practice, the prevalence of hand injuries is 10 for each 1000 patients per year, while the prevalence for wrist injuries is 6 for each 1000 patients per year.^4^ In an ED, injuries to the hand and wrist are common and they account for between 10% and 30% of all presentations.^3 5–7^ Traumatic hand injuries are a frequent part among work-related injuries and can result in prolonged sick leave. They represent a considerable economic burden, with both high healthcare and productivity costs.^5^ If not treated properly, patients may experience lifelong pain and functional limitations that have major effects on the quality of life and could result in patients losing their jobs.^8^

The standard diagnostic work-up for non-chronic finger, hand and wrist trauma consists of history taking, a physical examination and, if needed, imaging. There is general agreement that a detailed patient history and a conscientious clinical examination should be standard methods of diagnosing wrist pain.^9^ Nevertheless, the diagnosis of wrist pathologies remains complex and challenging, since the wrist contains many joints that function together to move the hand, and there is increasing demand for evidence for diagnostic technologies, such as imaging tools.^10^

Evidence-based medicine is required to create well-founded policies for non-chronic finger, hand and wrist ligament and tendon injuries. It is essential to distinguish between diagnosing these injuries in hospital care and in non-institutionalised GP care, as results from diagnostic studies in hospital care cannot automatically be translated into guidelines for non-institutionalised GP care.^11^ Diagnostic accuracy is affected by the prevalence of the pathology. Predictive values are largely dependent on the prevalence of the pathology in the examined population. Therefore, predictive values from one study should not be transferred to another setting with a different prevalence of the disease in the population.^12^ Nevertheless, currently available systematic reviews on the diagnostic accuracy of tests for the diagnosis of finger, hand and wrist pathologies did not distinguish between hospital and non-institutionalised GP care settings when presenting their results.^10 13–15^ Within the available systematic reviews, published up to 2015, no studies were found on the diagnostic accuracy of history taking and only the scaphoid shift test and high-resolution MRI were recommended for diagnosing triangular fibrocartilage complex (TFCC) tears.^10 13–15^

The main purpose of the present study was to provide a systematic overview of the diagnostic accuracy of history taking, physical examination and imaging for detecting non-chronic ligament and tendon injuries of the finger, hand and wrist. The secondary aim of this study was to retrieve the clinical care setting (hospital or non-institutionalised GP) of the eligible studies and the studies published in previous systematic reviews.

## Methods

The Preferred Reporting Items for Systematic Reviews and Meta-Analyses statement was used to guide the conduct and reporting of the study.^16^ A review protocol was composed prior to searching the literature, but central registration was not completed.

### Search strategy

A biomedical information specialist (Wichor M Bramer) from the Medical Library at Erasmus MC performed a search for studies in Medline, Embase, Cochrane Library, Web of Science, Google Scholar ProQuest and Cinahl from 2000 up to 6 February 2019. This starting point was used since multiple reviews are available that already cover the period up to the year 2000 (table 1). Search terms included hand, finger and wrist injuries, history taking, provocative test(s), diagnostic test(s) and imaging tests. The full electronic search strategy for the Embase database is presented in online supplemental appendix 1.

10.1136/bmjopen-2020-037810.supp1 Supplementary data

**Table 1:** Characteristics of the eligible studies (N=23)

Author (year)	Participants	Design	Setting (country)	Trauma	Index test 1	Index test 2	Reference test
Wrist injuries							
Anderson *et al* (2008)^23^	102	Retrospective	Not described (USA)	TFCC/SLIL /LTIL/UTIL	MRI (1.5 T)	MRI (3 T)	Arthroscopy
Pahwa *et al* (2014)^32^	53	Prospective	Not described (India)	TFCC/SLIL/LTIL	MRI (1.5 T)	MR arthrography	Arthroscopy
Prosser *et al* (2011)^33^	105	Prospective	Private hand clinic (Australia)	TFCC/SLIL/LTIL	MRI (1 T)	Provocative tests	Arthroscopy
Langner *et al* (2015)^40^	38	Not described	Not described (Germany)	SL dissociation	Cine MRI (3 T)	Cineradiography	Arthroscopy
Spaans *et al* (2013)^41^	37	Not described	Department for hand and plastic surgery* (The Netherlands)	SLIL (complete tear)	MRI (3 T)		Arthrotomy
Greditzer *et al* (2016)^24^	26	Retrospective	Department for hand and plastic surgery* (USA)	SLIL	MRI (1.5 T) axial sequences	MRI (1.5 T) coronal sequences	Arthroscopy
Al-Hiari (2013)^34^	42	Prospective	Orthopaedic surgery* (Jordan)	TFCC (full-thickness tears)	MR arthrography		Arthroscopy
Schmauss *et al* (2016)^25^	908	Retrospective	Department for hand and plastic surgery (Germany)	TFCC	MRI (resolution not described)	Provocative tests	Arthroscopy
Lee *et al* (2016)^35^	39	Prospective	Not described (China)	TFCC (full-thickness tears)/SLIL/LTIL	MR (3 T) arthrography without traction	MR (3 T) arthrography with traction	Conventional arthrography
Finlay *et al* (2004)^26^	26	Retrospective	Not described (Canada)	TFCC/SLIL/LTIL	US (9–13 MHz)		MR arthrography†
Dornberger*et al* (2015)^36^	72	Prospective	Hand surgery* (Germany)	SLIL	Radiographs		Arthroscopy
Koskinen *et al* (2012)^42^	52	Not described	Not described (Finland)	TFCC/SLIL/LTIL	CBCT arthrography		MR arthrography
Boer *et al* (2018)^27^	150	Retrospective	Plastic or orthopaedic surgery (The Netherlands)	TFCC	MRI (1.5 T or 3.0 T)	MR arthrography (1.5 or 3.0 T)	Arthroscopy
Lee and Yun (2018)^31^	65	Prospective	ED (Korea)	TFCC	US		MRI (3.0 T)
Suojärvi *et al* (2017)^37^	21	Prospective	Hand surgery (Finland)	SLIL/LTIL/TFCC	CBCT arthrography	MR arthrography	Arthroscopy
Mahmood *et al* (2012)^30^	30	Retrospective	General hospital (UK)	SLIL/LTIL/TFCC	MR arthrography		Arthroscopy
Hand and finger injuries							
Lutsky *et al* (2014)^28^	20	Retrospective	Not described (USA)	Collateral ligament tears of the MPJ of the fingers	MRI (open,1.5 T and 3 T)		Surgical findings
Guntern *et al* (2007)^29^	8	Retrospective	Not described (Switzerland)	A2 pulley lesion	Clinical examination		MRI (3 T)
Klauser *et al* (2002)^43^	64	Not described	Not described (Austria)	Finger pulley injuries	US (12 MHz)		MRI (1.5 T)
Lee *et al* (2000)^44^	10	Not described	Not described (USA)	Flexor tendon injuries	US (L10–5 MHz)		Surgical findings
Zhang *et al* (2012)^45^	92	Not described	Department of surgery (China)	Flexor tendon injuries	US (10 MHz)		Surgical findings
Mahajan *et al* (2016)^39^	30	Prospective	Emergency room and outpatients clinic of surgery and orthopaedics (the Netherlands)	UCL injuries	Clinical examination		MRI (1.5 T)
Shekarchi *et al* (2017)^38^	20	Prospective	ED (Iran)	UCL of the thumb	US		MRI

*Setting for the study was obtained after email contact.

†Tricompartment wrist arthrography.

CBCT, cone-beam CT; ED, emergency department; LTIL, lunotriquetral interosseous ligament; MPJ, metacarpophalangeal joint; MR, magentic resonance; SLIL, scapholunate interosseous ligament; TFCC, triangular fibrocartilage complex; UCL, ulnar collateral ligament; US, ultrasonography; UTIL, ulnotriquetral interosseous ligament.

### Study selection criteria

Studies describing diagnostic accuracy of history taking, physical examination or imaging in adult patients (age ≥16 years) with non-chronic finger, hand and wrist ligament and tendon injuries were included. Diagnostic accuracy was rabeported or could be calculated. Case reports, reviews and conference proceedings were excluded. Distal radius and ulna injuries were also excluded. Chronic injuries (eg, osteoarthritis) were excluded as a result of another pathophysiology. There was no gold-standard reference test against which to assess history taking, physical examination or imaging measurements. Surgical observations (arthroscopy) are the reference standards for confirming a diagnosis of non-chronic hand, finger or wrist injury, although only a subset of patients suspected of having non-chronic hand, finger or wrist injury require surgery. To decrease verification bias, diagnostic-imaging techniques for non-chronic hand injury were accepted as reference tests as well. Since tendinopathy does not typically require surgery, imaging is also a pragmatic reference standard for this condition. As this review focused on non-chronic pathologies, studies, including patient with chronic pathologies (eg, osteoarthritis and rheumatic arthritis), were excluded. Infection and neurological injuries are out of the scope of this review and are, therefore, not included. Carpal tunnel syndrome is extensively described in the literature and was, therefore, not included in this review.^17–19^ Diagnoses of musculoskeletal soft-tissue tumours were also excluded. No language restrictions were applied. For languages of the eligible studies other than English, Google translate was used for the first translation of these studies. If necessary, a professional translator was consulted.^20^

Two reviewers (PK and Yassine Aaboubout) read all titles and abstracts independently. Articles that could not be excluded on the basis of the title and/or abstract were retrieved in full text and were read and checked for inclusion by the two reviewers independently. If there was no agreement, a third reviewer (JR) made the final decision. In addition, the reference lists of all included studies were reviewed to check for additional relevant studies.

### Data collection process and methodological quality assessment

In the current review, our primary outcome measures were the positive predictive value (PPV) and the negative predictive value (NPV) of diagnostic tests. Secondary outcome measure were the sensitivity (Se), specificity (Sp) and accuracy of diagnostic tests.

Two reviewers (PK and JR) independently extracted the data. Data were extracted describing the study design, characteristics of the study population, test characteristics, setting (hospital care or non-institutionalised GP care) and diagnostic parameters. The following values were extracted, when documented: Se, Sp, accuracy, PPV and NPV. If diagnostic parameters were not reported, they were calculated from reported data or authors were contacted by email when data were unavailable. The following formula was used, when calculating diagnostic accuracy: diagnostic accuracy=(the number of true positives+the number of true negatives)/total number of subjects.^21^ If an included study presented results from multiple independent observers, accuracy measures were averaged over the observers. Furthermore, data of the studies published in previous systematic reviews were extracted describing the setting (hospital care or non-institutionalised GP care). Methodological quality was assessed using the Quality Assessment of Diagnostic Accuracy Studies 2 (QUADAS-2) checklist.^22^ This tool allows more transparent rating of bias and applicability in primary diagnostic accuracy studies. The QUADAS-2 tool consists of four domains: patient selection, index test, reference standard, and flow and timing. Two reviewers (PK and JR) independently assessed the risk of bias and applicability of each included study. Disagreements were resolved by discussion. Questions were answered with ‘yes’, ‘no’ or ‘unclear’.

### Patient and public involvement

Patients and members of the public were not involved in this systematic review update.

## Results

### Study selection

**Figure 1:**
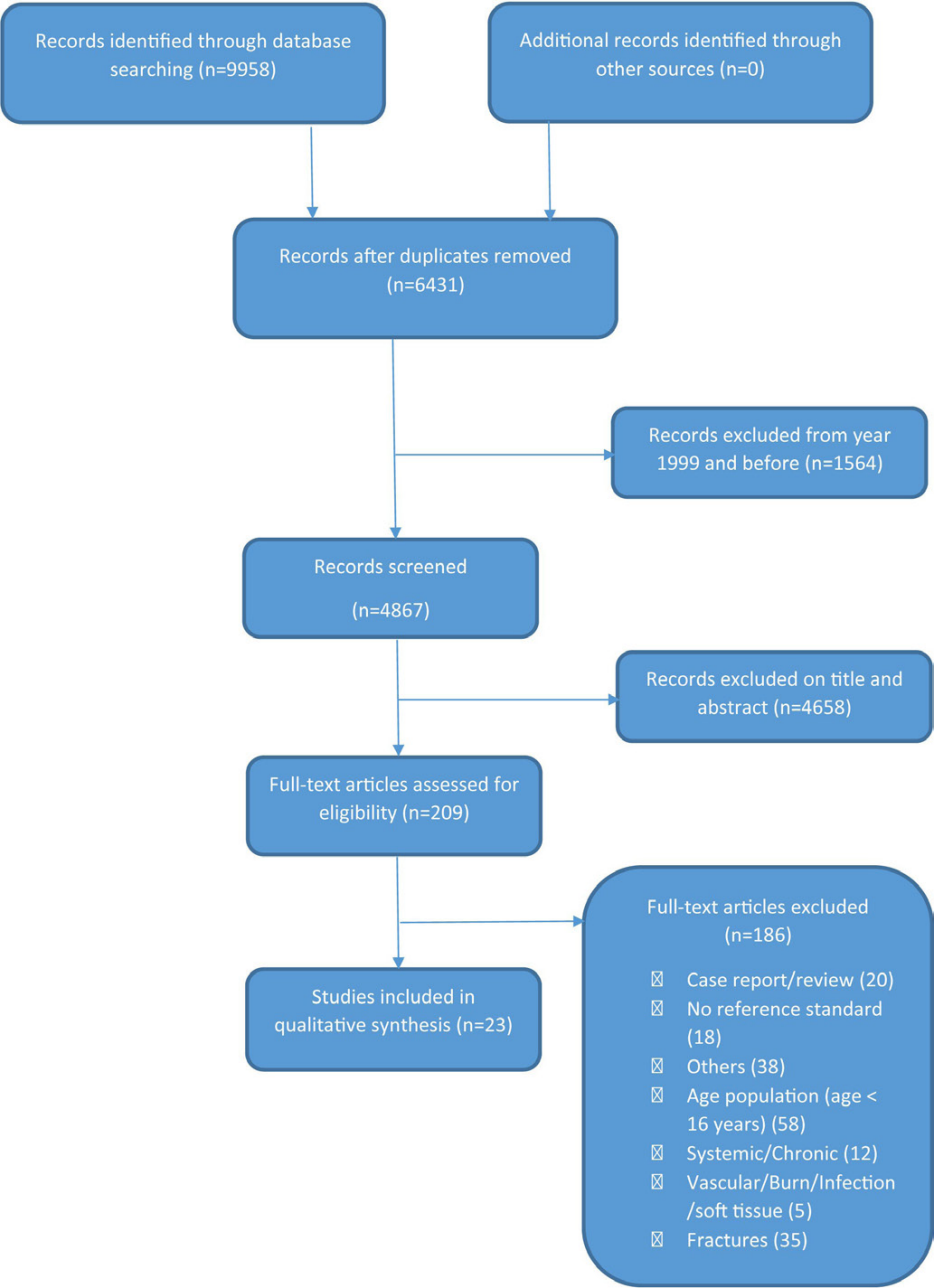
Flow chart study selection.

The flow diagram for the categorisation process is presented in figure 1. We assessed 209 full-text articles for eligibility out of 4867 records identified through database searches. A total of 23 diagnostic studies were finally identified, assessed and interpreted.

### Study characteristics

The characteristics of the studies are presented in table 1.

Eight studies were retrospective^23–30^ nine studies were prospective^31–39^ and six studies^40–45^ gave no description of the study design. Eight studies^23 25 27 31 33 36 43 45^ included more than 60 participants; six of these studies^23 25 27 31 33 36^ described wrist pathologies and two^43 45^ described hand pathologies. In total, 16 studies^23–27 30–37 40–42^ described injuries to the wrist anatomy and seven studies^28 29 38 39 43–45^ described injuries to the hand/finger anatomy.

### Quality assessment

There was considerable underreporting of important quality domains in most studies (see table 2).

**Table 2:** Summary of methodological quality according to Quality Assessment of Diagnostic Accuracy Studies 2

Author (year), index test(s)	Risk of bias				Applicability concerns		
	Patient selection	Index test	Reference standard	Flow and timing	Patient selection	Index test	Reference standard
Wrist disabilities							
Anderson *et al* (2008)^23^	LR	LR	HR	LR	LR	LR	LR
Pahwa *et al* (2014)^32^	UR	LR	HR	HR	LR	LR	LR
Prosser *et al* (2011), provocative tests^33^	LR	LR	LR	LR	LR	LR	LR
MRI	LR	LR	HR	LR	LR	LR	LR
Langner *et al* (2015)^40^	LR	LR	HR	HR	LR	LR	LR
Spaans *et al* (2013)^41^	UR	LR	LR	UR	LR	LR	LR
Greditzer *et al* (2016)^24^	HR	LR	HR	LR	LR	LR	LR
Al-Hiari (2013)^34^	LR	LR	HR	LR	LR	LR	LR
Schmauss *et al* (2016)^25^	LR	HR	HR	LR	LR	LR	LR
Lee *et al* (2016)^35^	LR	HR	HR	LR	LR	LR	LR
Finlay *et al* (2004)^26^	UR	LR	LR	LR	LR	LR	LR
Dornberger *et al* (2015)^36^	LR	LR	HR	LR	LR	LR	LR
Koskinen *et al* (2012)^42^	LR	HR	HR	LR	LR	LR	LR
Boer *et al* (2018)^27^	HR	UR	HR	LR	LR	LR	LR
Lee and Yun (2018)^31^	LR	LR	LR	LR	LR	LR	LR
Suojärvi *et al* (2017)^37^	LR	LR	HR	HR	LR	LR	LR
Mahmood *et al* (2012)^30^	UR	LR	UR	LR	LR	LR	LR
Hand and finger disabilities							
Lutsky *et al* (2014)^28^	LR	UR	HR	LR	LR	LR	LR
Guntern *et al* (2007)^29^	LR	HR	LR	LR	LR	LR	LR
Klauser *et al* (2002)^43^	LR	HR	LR	HR	LR	LR	LR
Lee *et al* (2000)^44^	UR	LR	LR	LR	LR	LR	LR
Zhang *et al* (2012)^45^	LR	LR	HR	LR	LR	LR	LR
Mahajan *et al* (2016)^39^	LR	LR	LR	LR	LR	LR	LR
Shekarchi *et al*(2017)^38^	UR	LR	LR	LR	LR	LR	LR

HR, high risk; LR, low risk; U, unclear risk.

Two studies had low risk of bias on all quality domains.^31 33^ In 8^24 26 27 30 32 38 41 44^ of the 23 studies, patient selection was not well documented. Furthermore, the risk of bias was predominantly influenced by the lack of a proper description of the index test (30%, 7/23)^25 27–29 35 42 43^ or the reference standard (65%, 15/23).^23–25 27 28 30 32–37 40 42 45^ Regarding flow and timing, not all patients received the reference standard in four studies (22%, 5/23).^32 34 37 41 43^ Due to our selection procedure, all the studies match the review question.

### Accuracy of diagnostic tests concerning wrist injuries

None of the studies evaluated the diagnostic accuracy of history taking. Physical examination was evaluated in two studies for diagnosing lesions of the TFCC.^25 33^ Provocative wrist tests for diagnosing scapholunate interosseous ligament (SLIL) and lunotriquetral interosseous ligament (LTIL) lesions was assessed in one study.^33^

Radiographs were used as an index test in one study for diagnosing SLIL lesions.^36^ Ultrasonography (US) for diagnosing TFCC lesions was used in two studies.^26 31^ Two studies used cone-beam CT (CBCT) as index for diagnosing TFCC lesions.^37 42^ In 12 studies, MRI was used as an index test.^23–25 27 30 32–35 37 40 41^ The accuracy of MRI for TFCC, SLIL, LTIL and ulnotriquetral interosseous ligament (UTIL) lesions with high-resolution (3 T) techniques ranging from 89% to 91%, 75% to 92%, 91% and 100%, respectively. The accuracy of MRI for TFCC, SLIL, LTIL and UTIL lesions with low-resolution (1.5 T) techniques ranging from 81% to 100%, 67% to 81%, 81% to 94% and 95%, respectively. The accuracy measures of the diagnostic tests are presented in table 3.

**Table 3:** Accuracy of the diagnostic tests of the wrist

Author (year)	Index test 1	Reference test	Trauma	Se (%) (95% CI)	Sp (%) (95% CI)	Accuracy (%) (95% CI)	PPV (%) (95% CI)	NPV (%) (95% CI)
Physical examination								
Prosser *et al*^33^ (2011)	Provocative tests	Arthroscopy	TFCC	58	69	73	71	55
			SLIL	61	79	78	68	74
			LTIL	17	84	95	6	94
Schmauss *et al*^25^ (2016)	Fovea sign	Arthroscopy	TFCC	73	44	58	53	66
	Ulna grinding test			90	20	56	54	65
Imaging: radiographs								
Dornberger *et al*^36^ (2015)	Radiographs (Stecher’s projection)	Arthroscopy	SLIL	(76.9+80.8)/2*	(86.4+84.1)/2*	(92.7+90.6)/2*	(76.9+75)/2*	(86.4+88.1)/2*
Imaging: US								
Finlay *et al*^26^ (2004)	US (9–13 MHz)	MR arthrography tricompartment	SLIL	100	100	100	100	100
			TFCC	64	100	85	100	79
			LTIL	25	100	77	100	75
Lee and Yun^31^ (2018)	US	MRI	TFCC, total	99*	88*	97*	97*	95*
Imaging: MRI								
Anderson *et al*^23^ (2008)	MRI (1.5 T)	Arthroscopy	TFCC	82	59	83 (72.4 to 89.9)†		
			SLIL	57	83	78 (67.2 to 86.3)†		
			UTIL	57	89	95 (86.1 to 98.3)†		
			LTIL	22	94	86 (75.3 to 91.9)†		
	MRI (3 T)		TFCC	90	74	91 (75.8 to 96.8)†		
			SLIL	70	94	91 (75.8 to 96.8)†		
			UTIL	67	87	100 (97.9 to 100)†		
			LTIL	50	94	91 (75.8 to 96.8)†		
Pahwa *et al*^32^ (2014)	MR arthrography	Arthroscopy	TFCC	100	100	100	100	100
			SLIL	100	100	100	100	100
			LTIL	100	100	100	100	100
	MRI (1.5 T) MEDIC		TFCC	83	100	81	91	60
			SLIL	63	100	81	100	73
			LTIL	40	100	81	100	73
	MRI FS PD/T2		TFCC	75	100	75	90	50
			SLIL	38	100	69	100	62
			LTIL	20	100	75	100	73
Prosser *et al*^33^ (2011)	MRI (1 T)	Arthroscopy	TFCC			86 (PT+MRI)		
			SLIL			80 (PT+MRI)		
			LTIL			94 (PT+MRI)		
Schmauss *et al*^25^ (2016)	MRI resolution not described	Arthroscopy		76	41	58	55	65
Langner *et al*^40^ (2015)	Cine MRI (3.0 T) and cineradiography	Arthroscopy	SL dissociation	85	90	92		
Spaans *et al*^41^ (2013)	MRI (3 T)	Arthrotomy	SLIL	75.5*	100†	75*	98.5*	8†
Greditzer *et al*^24^ (2016)	MRI (1.5 T) axial sequences	Arthroscopy	SLIL	79	82	80	76	84
	MRI (1.5 T) coronal sequences		SLIL	65	69	67	68	71
Al-Hiari^34^ (2013)	MR arthrography	Arthroscopy	TFCC	93	80	85		
Lee^35^ (2016)	MR arthrography without traction	Conventional arthrography	TFCC	83	81	83	87	76
			SLIL	66	97	95	67	97
			LTIL	57	94	88	67	91
	MR arthrography with traction		TFCC	96	100	98	100	94
			SLIL	100	100	100	100	100
			LTIL	100	100	100	100	100
Boer *et al*^27^ (2018)	MRI (1.5 T)	Arthroscopy	TFCC	71	75	100	71	75
	MRI (3.0 T)	Arthroscopy	TFCC	73	67	89	83	52
	MR arthrography (1.5 T)	Arthroscopy	TFCC	80	100	80	100	50
	MR arthrography (3.0 T)	Arthroscopy	TFCC	73	100	73	100	60
Suojärvi *et al*^37^ (2017)	MR arthrography	Arthroscopy	SLIL	25 (3 to 65)	80 (61 to 92)	68 (51 to 83)	25 (3 to 65)	80 (61 to 92)
			LTIL	50 (7 to 93)	77 (59 to 90)	74 (57 to 88)	22 (3 to 60)	92 (75 to 99)
			TFCC	44 (22 to 69)	50 (25 to 75)	47 (30 to 65)	50 (25 to 75)	44 (21 to 69)
			SLIL or LTIL	33 (7 to 60)	79 (67 to 88)	72 (56 to 82)	24 (7 to 50)	86 (74 to 94)
Mahmood *et al*^30^ (2012)	MR arthrography	Arthroscopy	SLIL	91	88		83	88
			LTIL	100	100		100	100
			TFCC	90	75		85	80
Imaging: CT								
Koskinen *et al*^42^ (2012)	CBCT arthrography	MR arthrography	TFCC	76	90	87	83	87
			SLIL	56	91	83	67	89
			LTIL	83	81	82	44	96
Suojärvi *et al*^37^ (2017)	CBCT	Arthroscopy	SLIL	63 (24 to 91)	87 (69 to 96)	82 (66 to 92)	56 921 to 86)	90 (73 to 98)
			LTIL	100 (40 to 100)	59 (41 to 76)	64 (46 to 79)	24 (7 to 50)	100 (83 to 100)
			TFCC	67 (40 to 87)	89 (63 to 98)	77 (60 to 90)	86 (57 to 98)	73 (50 to 89)
			SLIL or LTIL	75 (43 to 95)	76 (65 to 86)	73 (61 to 83)	35 (16 to 53)	95 (86 to 99)

*Average between presented individual values of two readers.

†Only reported for one of two readers.

CBCT, cone-beam CT; FS, fat suppressed; LTIL, lunotriquetral interosseous ligament; MEDIC, multiple-echo data image combination; MR, magnetic resonance; n/a, not available due to low prevalence; NPV, negative predictive value; PD/T2, proton density/tesla2; PPV, positive predictive value; PWT, provocative wrist tests; Se, sensitivity; SLIL, scapholunate interosseous ligament; Sp, specificity; TFCC, triangular fibrocartilage complex; US, ultrasonography; UTIL, ulnotriquetral interosseous ligament.

In addition to the data presented in table 3, the study of Schmauss *et al* presented the diagnostic accuracy of their tests separately for different subgroups.^25^ These results are summarised in online supplemental appendix 2.

10.1136/bmjopen-2020-037810.supp2 Supplementary data

### Accuracy of diagnostic tests concerning hand and finger injuries

Table 4 describes the accuracy of the diagnostic tests for non-chronic hand and finger injuries.^28 29 38 39 43–45^

**Table 4:** Accuracy of the diagnostic tests of the hand and fingers

Author (year)	Index test 1	Reference test	Trauma	Se (%) (95% CI)	Sp (%) (95% CI)	Accuracy (%) (95% CI)	PPV (%) (95% CI)	NPV (%) (95% CI)
Lutsky *et al*^28^ (2014)	MRI (open,1.5 T or 3 T)	Surgical findings	Collateral ligament tears of the MPJ of the fingers	64	∞	64	100	∞
Guntern *et al*^29^ (2007)	Clinical examination	MRI (3 T)	A2 pulley lesion	88	100	88	100	95
Klauser *et al*^43^ (2002)	US (12 MHz)	MRI (1.5 T) (and surgical findings, n=7)	Finger pulley injuries	98	100	99	100	97
Lee *et al*^44^ (2000)	US (10–5 MHz)	Surgical findings	Flexor tendon injuries			90		
Zhang *et al*^45^ (2012)	US (10 MHz)	Surgical findings	Flexor tendon injuries			100		
	History and clinical examination					34		
Mahajan *et al*^39^ (2016)	Clinical examination	MRI (1.5 T)	UCL injuries	91	75	87	91	75
Shekarchi *et al*^38^ (2017)	US	MRI	UCL of the thumb	71 (30 to 95)	85 (54 to 97)	80	71 (30 to 95)	85 (54 to 97)

MPJ, metacarpophalangeal joint; NPV, negative predictive value; PPV, positive predictive value; Se, sensitivity; Sp, specificity; UCL, ulnar collateral ligament; US, ultrasonography.

Two studies concerned flexor tendon injuries,^44 45^ while the other studies concerned collateral ligament tears of the metacarpophalangeal joint of the fingers,^28^ A2 pulley lesions,^29^ finger pulley injuries^43^ and ulnar collateral ligament (UCL) injuries.^38 39^ None of the studies involved history taking. Clinical examination was used three times as an index test.^29 39 45^ The Se, Sp, accuracy, PPV and NPV of physical examination in hand and finger injuries ranged from 88% to 99%, 75% to 100%, 34% to 88%, 91% to 100% and 75% to 95%, respectively. MRI was used once as an index test.^28^ Four studies used ultrasonography (US) as an index test.^38 43–45^ The accuracy of US in flexor injuries ranged from 90% to 100%.^44 45^ The accuracy of US for finger pulley injuries and UCL of the thumb was 99% and 80%, respectively.^38 43^

### Clinical care setting

The clinical care setting was described in 9 out of 23 studies and was obtained by contacting the authors for an additional 4 studies: a private hand clinic,^33^ ED,^31 38^ department for hand and plastic surgery,^24 27 34 36 41^ surgery,^45^ orthopaedics department^27 34^ and in an emergency room and outpatient clinic of a surgery and orthopaedics department.^39^ Despite multiple attempts to contact the authors by email, clarification regarding the setting could not be obtained for the remaining 10 studies.

## Discussion

The standard diagnostic work-up for non-chronic finger, hand and wrist trauma consists of history taking, a physical examination and, if needed, imaging. There is general agreement that a detailed patient history and a conscientious clinical examination should be standard methods of diagnosing wrist pain.^9^ Our systematic review showed that there is still a gap in knowledge regarding valid diagnostic tests for non-chronic wrist ligament and tendon injuries. Moreover, for the first time, the lack of high-quality evidence for the diagnosis of ligament and tendon injuries in the hand and fingers has been highlighted in the current systematic overview of the literature.

Previous reviews showed that a high-resolution MRI was an accurate means for diagnosing TFCC tears and an MRI was slightly specific for tears of the intrinsic ligament, but its sensitivity is low.^10 14^ Current review showed that the accuracy measures for an MRI showed a wide range in diagnostic outcome values, with diagnostic accuracy measures no better for a high-resolution MRI. The present results indicate that the accuracy for tears of the TFCC, SLIL and LTIL is increased by magnetic resonance arthrography (MRA).

### Diagnostic accuracy of the diagnostic tests of the wrist

Although a common practice in hospital care, in previous reviews^10 13–15^ and in current systematic review update, no studies were identified on the diagnostic accuracy of history taking for non-chronic ligament and tendon injuries of the wrist.

This systematic review update included one new study on physical examinations for diagnosing non-chronic ligament and tendon injuries of the wrist, which did not affect the previous conclusion that physical examination is of limited value for diagnosing non-chronic ligament and tendon injuries of the wrist.^25^

In previous reviews, only the diagnostic performance for MRI and/or MRA of the wrist were examined. This showed that the accuracy of MRI diagnoses of tears of the TFCC was fairly satisfactory (PPV ranged from 71% to 100% and NPV ranged from 37% to 90% for TFCC, PPV ranged from 25% to 100% and NPV ranged from 72% to 94% for SL ligament and PPV ranged from 0% to 100% and NPV ranged from 74% to 95% for LT ligament) and the best with high-resolution techniques. Contrary, Se, Sp and accuracy were low for diagnosing intrinsic carpal ligaments injuries (SL and LT), using high-resolution techniques.^10 14^ MRA, rather than MRI, was recommended to be used in daily practise for the diagnosis of TFCC injuries.^10 15^ In the current review, the accuracy measures for an MRI showed a wide range in diagnostic outcome values, with diagnostic accuracy measures no better for imaging at 3 T than at 1.5 T. As previously shown for full-thickness TFCC injuries, the present results indicate that the accuracy for tears of the TFCC, SLIL and LTIL is increased by MRA.^15^ CT arthrography is an alternative in patients when an MRI is contraindicated or when an MRI is not available.^42^

In the current review, five studies used another imaging tool, namely, radiograph,^36^ US^26 31^ and CBCT^37 42^ for diagnosing non-chronic ligament and tendon injuries of the wrist. The diagnostic accuracy of radiograph was limited. Examination of SLIL and TFCC with US showed promising results and the added value should be further explored. Based on the included studies, CBCT has no added value in assessing non-chronic ligament and tendon injuries of the wrist, especially when we take the methodological quality of the studies into account.

However, a dynamic four-dimensional CT for the detection of SLIL or LTIL injuries is promising.^46 47^ Nevertheless, the diagnostic accuracy has not yet been studied. At present, there is still insufficient scientific evidence regarding the ideal imaging technique for non-chronic intrinsic carpal ligament injuries of the wrist.

In the current systematic review update and previous systematic reviews, the reported diagnostic accuracy measures for imaging modalities were characterised by markedly heterogeneous results. It was not appropriate to pool results for the diagnostic accuracy of imaging, due to a lack of multiple imaging studies on one specific wrist injury. Based on previews and the current review, we can conclude that an MRA rather than an MRI is the preferred imaging tool in hospital care setting for detecting non-chronic ligament and tendon injuries of the wrist. The current review focused on diagnostic tests and not on the treatment options for wrist complaints. Arthroscopy, as diagnostic tool, was one of the reference standards in this systematic review. In our opinion, it is essential that readily accessible and relatively inexpensive, non-invasive diagnostics are available to and are preferred by clinicians. For some wrist complaints, arthroscopy may be the preferred diagnostic option. However, it is more expensive and invasive than an MRI. For that reason, diagnostic arthroscopy should be applied with caution, unless a patient is suspected of having non-chronic hand, finger or wrist injury and require therapeutic intervention. The advantage of arthroscopy above MRI is the dynamic modality.

### Diagnostic accuracy of the diagnostic tests of the hand and the fingers

According to our knowledge, there are no reviews previously published to date on the diagnostic accuracy of history taking, physical examination and imaging for non-chronic ligament and tendon injuries of the finger and hand.

We identified three studies on the diagnostic accuracy of history taking and/or clinical examination.^29 39 45^ One study^39^ had no methodological limitation, while the other two studies had methodological flaws (high risk of bias) on index test^29^ and reference standard.^45^ In addition, each study evaluated different diagnostics tests for different pathologies. So there is limited evidence on the diagnostic accuracy of history taking and physical examination for diagnosing hand and finger injuries.

Imaging studies examined a wide variety of imaging tools and pathologies. Moreover, studies with imaging tools as a diagnostic modality had methodological flaws and serious limitations, so we have to interpret these results with caution. Only the study of Lee *et al* had relatively few methodological flaws.^44^ These authors showed that US can possibly help to evaluate completely lacerated flexor tendon injuries. Nevertheless, as indicated by the authors, US cannot accurately determine the status of partially transected tendons.^44^ The reported diagnostic accuracy measures for imaging modalities were characterised by markedly heterogeneous results. It was not appropriate to pool results for the diagnostic accuracy of imaging, due to the limited number of studies on one specific hand or finger injury and because of the diversity among the eligible studies.

### Clinical care setting

The secondary aim of this study was to include the clinical care setting (hospital or non-institutionalised GP) of the eligible studies and the studies published in previous systematic reviews. We assume that all studies included in the current and previous reviews were done in a hospital care setting; this was either described in the paper, was confirmed by the authors or due to the fact that all authors of the remaining studies were only affiliated to hospitals.

It is essential to distinguish between diagnosing these injuries in hospital care and in non-institutionalised GP care, as results from diagnostic studies in hospital care cannot automatically be translated into guidelines for non-institutionalised GP care.^11^ Since previous systematic reviews and the current update of the literature did not identify any studies performed in non-institutionalised GP care, it is not possible to advise GPs with certainty based on the available evidence. Given the burden of non-chronic hand and wrist trauma in non-institutionalised GP care, diagnostic studies focusing on non-chronic hand, finger and wrist ligament and tendon injuries are urgently needed.^1 2^

## Conclusions

Our systematic review showed that there is still a gap in knowledge regarding valid diagnostic tests for non-chronic wrist ligament and tendon injuries. For the first time, the lack of high-quality evidence for the diagnosis of ligament and tendon injuries in the hand and fingers has been highlighted. Although some imaging modalities seemed to be acceptable for the diagnosis of ligament and tendon injuries in the wrist in patients presenting to secondary care, there are limited tools for adequate diagnosis available to GPs. If not diagnosed and treated properly, patients may experience lifelong pain and functional limitations that have major effects on the quality of life and could result in patients losing their jobs.

## Supplementary Material

Reviewer comments

Author's manuscript
