# Supplementary material for: Diagnostic accuracy of history taking, physical examination and imaging for non-chronic finger, hand and wrist ligament and tendon injuries: a systematic review update
Source: BMJ Open. 2020 Nov 5;10(11):e037810. doi: 10.1136/bmjopen-2020-037810 (PMC7646346; doi:10.1136/bmjopen-2020-037810)
Supplement: Supplementary data [file bmjopen-2020-037810supp001.pdf]

Appendix 1

Database	Search terms
Embase	('hand injury'/exp OR 'wrist injury'/exp OR 'wrist fracture'/exp OR (('hand bone'/exp OR wrist/exp OR hand/exp OR 'wrist pain'/exp OR 'hand pain'/exp) AND ('bone injury'/exp OR fracture/de OR 'ligament injury'/exp OR 'ligament rupture'/exp)) OR (((hand OR hands OR wrist* OR finger* OR carpal* OR carpus OR phalanx* OR metacarp* OR capitate* OR hamat* OR lunat* OR pisiform* OR scaphoid* OR trapezium* OR trapezoid* OR triquetr* OR navicular* OR lunar OR semilunar* OR multangulum* OR pyramid* OR metacarpophalang* OR thumb* OR 'distal radius' OR 'distal ulna' OR 'distal radial' OR 'distal ulnar' OR scapholunate* OR lunotriquetral* OR 'triangular fibrocartilaginous' OR SLIL OR LTIL OR tfcc OR 'ulnar collateral ligament' OR 'ulnar collateral ligaments' OR ucl) NEAR/3 (injur* OR trauma* OR wound* OR lesion* OR dislocate* OR fracture* OR damage* OR tear* OR sprain* OR displace* OR rupture*))):ab,ti) AND ('diagnostic test'/de OR 'function test'/exp OR 'diagnostic error'/exp OR 'diagnostic accuracy'/exp OR 'diagnostic value'/exp OR 'differential diagnosis'/exp OR 'delayed diagnosis'/exp OR 'sensitivity and specificity'/exp OR (((diagnos* OR detect* OR differen* OR strength* OR motion*) NEAR/3 (test* OR accura* OR error* OR false OR fail* OR value* OR impact* OR effective* OR earl* OR missed OR correct* OR incorrect* OR delay* OR difficult* OR negative* OR positive* OR sensitivit* OR specificit* OR confirm* OR abilit*)) OR (diagnos* NEAR/3 differen*) OR misdiagnos* OR underdiagnos* OR undetect* OR (predict* NEAR/3 value*) OR (function* NEAR/3 test*) OR (false NEAR/3 (negative* OR positive*))):ab,ti) NOT ([Conference Abstract]/lim OR [Letter]/lim OR [Note]/lim OR [Editorial]/lim) AND [english]/lim NOT ([animals]/lim NOT [humans]/lim)

Search terms for the other databases are available upon request.
