# Supplementary material for: Diagnostic accuracy of history taking, physical examination and imaging for non-chronic finger, hand and wrist ligament and tendon injuries: a systematic review update
Source: BMJ Open. 2020 Nov 5;10(11):e037810. doi: 10.1136/bmjopen-2020-037810 (PMC7646346; doi:10.1136/bmjopen-2020-037810)
Supplement: Supplementary data [file bmjopen-2020-037810supp002.pdf]

Appendix 2. Diagnostic Accuracy of Tests in Different Subgroups Presented in the Study of Schmauss et al.[26]

	Se (%)	Sp (%)	Accuracy (%)	PPV (%)	NPV (%)
Ulna carpal pain					
Ulnar fovea sign	88	9	54	56	39
Ulnar grinding test	93	12	58	58	58
MRI	88	23	58	57	61
Ulno carpal pain and prior trauma					
Ulnar fovea sign	90	8	54	55	39
Ulnar grinding test	93	12	57	57	57
MRI	93	17	59	58	65
Palmar A1 lesion					
Ulnar fovea sign	86	9	40	39	50
Ulnar grinding test	90	12	42	39	66
MRI	90	23	48	41	79
Palmer 2c Lesion					
Ulnar fovea sign	85	9	26	22	68
Ulnar grinding test	93	12	30	23	86
MRI	88	23	37	24	87
