# Supplementary material for: Diagnostic accuracy of history taking, physical examination and imaging for non-chronic finger, hand and wrist ligament and tendon injuries: a systematic review update
Source: BMJ Open. 2020 Nov 5;10(11):e037810. doi: 10.1136/bmjopen-2020-037810 (PMC7646346; doi:10.1136/bmjopen-2020-037810)
Supplement: Author's manuscript [file bmjopen-2020-037810.draft_revisions.pdf]

BMJ Open

BMJ Open is committed to open peer review. As part of this commitment we make the peer review history of every article we publish publicly available.

When an article is published we post the peer reviewers' comments and the authors' responses online. We also post the versions of the paper that were used during peer review. These are the versions that the peer review comments apply to.

The versions of the paper that follow are the versions that were submitted during the peer review process. They are not the versions of record or the final published versions. They should not be cited or distributed as the published version of this manuscript.

BMJ Open is an open access journal and the full, final, typeset and author-corrected version of record of the manuscript is available on our site with no access controls, subscription charges or pay-per-view fees (http://bmjopen.bmj.com).

If you have any questions on BMJ Open's open peer review process please email info.bmjopen@bmj.com

BMJ Open

Diagnostic accuracy of history taking, physical examination and imaging for non-chronic finger, hand and wrist ligament and tendon injuries: a systematic review update.

Journal:	*BMJ Open*
Manuscript ID	bmjopen-2020-037810
Article Type:	Original research
Date Submitted by the Author:	18-Feb-2020
Complete List of Authors:	Krastman, Patrick; Erasmus Medical Center,<br>Mathijssen, Nina; Reinier de Graaf Gasthuis, Orthopaedic<br>Bierma-Zeinstra, Sita; Erasmus University Medical Centre, Department of General Practice<br>Kraan, Gerald; Reinier de Graaf Hospital, Orthopeadic<br>Runhaar, Jos; Erasmus University Medical Center Rotterdam, Department of General Practice
Keywords:	Diagnostic radiology < RADIOLOGY & IMAGING, Hand & wrist < ORTHOPAEDIC & TRAUMA SURGERY, Adult orthopaedics < ORTHOPAEDIC & TRAUMA SURGERY

SCHOLARONE™
Manuscripts

I, the Submitting Author has the right to grant and does grant on behalf of all authors of the Work (as defined in the below author licence), an exclusive licence and/or a non-exclusive licence for contributions from authors who are: i) UK Crown employees; ii) where BMJ has agreed a CC-BY licence shall apply, and/or iii) in accordance with the terms applicable for US Federal Government officers or employees acting as part of their official duties; on a worldwide, perpetual, irrevocable, royalty-free basis to BMJ Publishing Group Ltd ("BMJ") its licensees and where the relevant Journal is co-owned by BMJ to the co-owners of the Journal, to publish the Work in this journal and any other BMJ products and to exploit all rights, as set out in our licence.

The Submitting Author accepts and understands that any supply made under these terms is made by BMJ to the Submitting Author unless you are acting as an employee on behalf of your employer or a postgraduate student of an affiliated institution which is paying any applicable article publishing charge ("APC") for Open Access articles. Where the Submitting Author wishes to make the Work available on an Open Access basis (and intends to pay the relevant APC), the terms of reuse of such Open Access shall be governed by a Creative Commons licence – details of these licences and which Creative Commons licence will apply to this Work are set out in our licence referred to above.

Other than as permitted in any relevant BMJ Author's Self Archiving Policies, I confirm this Work has not been accepted for publication elsewhere, is not being considered for publication elsewhere and does not duplicate material already published. I confirm all authors consent to publication of this Work and authorise the granting of this licence.

For peer review only

Diagnostic accuracy of history taking, physical examination and imaging for non-chronic finger, hand and wrist ligament and tendon injuries: a systematic review update.

Patrick Krastman MD[a], Nina M. Mathijssen PhD[c], Sita M.A. Bierma-Zeinstra PhD[a,b], Gerald Kraan PhD[c], Jos Runhaar PhD[a]

[a] Erasmus MC University Medical Center, Rotterdam, the Netherlands, Department of General Practice.

[b] Erasmus MC University Medical Center, Rotterdam, the Netherlands, Department of Orthopedics.

[c] Reinier de Graaf Groep, Delft, the Netherlands, Department of Orthopedic Surgery.

Patrick Krastman MD (corresponding author): wetenschap@dezorghoek.nl , +31(0)10-7044192

Nina M. Mathijssen PhD: N.Mathijssen@rdgg.nl , +31(0)15-2604863

Sita M.A. Bierma-Zeinstra PhD: s.bierma-zeinstra@erasmusmc.nl , +31(0)10-7044192

Gerald Kraan PhD: KraanG@rdgg.nl , +31(0)15-2604863

Jos Runhaar PhD: j.runhaar@erasmusmc.nl , +31(0)10-7044192

Keywords: wrist, hand, finger, diagnostic tests, traumatic injury, ligament.

For peer review only

Abstract

Objective: The diagnostic work-up for ligament and tendon injuries of the finger, hand and wrist consists of history taking, physical examination and imaging if needed, but the supporting evidence is limited. The main purpose of this study was to systematically update the literature for studies on the diagnostic accuracy of tests for detecting non-chronic ligament and tendon injuries of the finger, hand and wrist.

Methods: Medline, Embase, Cochrane Library, Web of Science, Google scholar ProQuest and Cinahl were searched from 2000 up to 6 February 2019 for identifying studies. Methodological quality was assessed using the QUADAS-2 checklist and sensitivity (Se), specificity (Sp), accuracy, positive predictive value (PPV) and negative predictive value (NPV) were extracted.

Results: None of the studies involved history taking. Physical examination, for diagnosing lesions of the triangular fibrocartilage complex (TFCC), showed Se, SP, accuracy, PPV and NPV ranging from 58-90%, 20-69%, 56-73%, 53-71% and 55-65%, respectively. Physical examination in hand and finger injuries the Se, Sp, accuracy, PPV and NPV ranged from 88 to 99%, 75 to 100%, 34 to 88%, 91 to 100% and 75 to 95%, respectively. The accuracy of Magnetic Resonance Imaging (MRI) with high-resolution (3 tesla) techniques for TFCC and interosseous ligaments of the proximal carpal row ranged from 89-91% and 75-100%, respectively. The accuracy of MRI with low-resolution (1.5 tesla) techniques for TFCC and interosseous ligaments of the proximal carpal row ranged from 81-100% and 67-95%, respectively.

Conclusions:

So far there is limited evidence on the diagnostic accuracy of history taking and physical examination. While MR Arthrography (MRA) rather than MRI is the preferred imaging tool for detecting non-chronic ligament and tendon injuries of the wrist, there is limited evidence

For peer review only - http://bmjopen.bmj.com/site/about/guidelines.xhtml

on which imaging tool is most accurate for diagnosing non-chronic ligament and tendon injuries of the hand and/or fingers.

Strengths and Limitations of this study:

- This is the first study that systematically reviewed the accuracy of diagnostic tests for non-chronic hand and finger injuries, next to previously described accuracy of diagnostic tests for non-chronic wrist injuries.
- Studies on wrist injuries published before 2000 were not evaluated and not included in the current systematic review, as these were adequately described in published systematic reviews.
- Diagnostic tests heterogeneity precluded meta-analysis.

For peer review only

For peer review only - http://bmjopen.bmj.com/site/about/guidelines.xhtml

Introduction

Wrist injuries are one of the most common presentations to the emergency department due to trauma and they commonly affect young people of working age.[1 2] In the Netherlands, 21 per cent of the patients initially consulted their general practitioner (GP) after a wrist injury, 41 per cent went directly to an outpatient clinic and 35 per cent had no further treatment.[3] Within the GP's practice, the prevalence of hand injuries is 10 for each 1000 patients per year, while the prevalence for wrist injuries is six for each 1000 patients per year.[4] In an Emergency Department (ED) injuries to the hand and wrist are common and they account for between 10% and 30% of all presentations.[3 5 6 7] Traumatic hand injuries are a frequent part among work-related injuries and can result in prolonged sick-leave. They represent a considerable economic burden, with both high health-care and productivity costs.[5] If not treated properly, patients may experience lifelong pain and functional limitations that have major effects on the quality of life and could result in patients losing their jobs.[8]

The standard diagnostic work-up for non-chronic finger, hand and wrist trauma consists of history taking, a physical examination and, if needed, imaging. There is general agreement that a detailed patient history and a conscientious clinical examination should be standard methods of diagnosing wrist pain.[9] Nevertheless, the diagnosis of wrist pathologies remains complex and challenging, since the wrist contains many joints that function together to move the hand, and there is increasing demand for evidence for diagnostic technologies, such as imaging tools.[10]

Evidence-based medicine is required to create well-founded policies for non-chronic finger, hand and wrist ligament and tendon injuries. It is essential to distinguish between diagnosing these injuries in hospital care and in non-institutionalized GP care, as results from diagnostic studies in hospital care cannot automatically be translated into guidelines for non-institutionalized GP care.[11] Nevertheless, currently available systematic reviews on the

For peer review only

diagnostic accuracy of tests for the diagnosis of finger, hand and wrist pathologies, as presented in Table 1, did not distinguish between hospital and non-institutionalized GP care settings when presenting their results.[10 12-14]

Table 1. Characteristics of the Currently Available Systematic Reviews on the Diagnostic Accuracy of Tests.

**Author, year**	**Population in eligible studies as described by the review authors**	**Diagnostic test**	**Trauma**	**Number of studies included**	**Pooled Se (95 % CI)**	**Pooled Sp (95 % CI)**	**Accuracy (95% CI)**	**PPV (95 % CI)**	**NPV (95 % CI)**	**+LR**	**-LR**	**Conclusion**
**HISTORY TAKING**												
No reviews identified												
**PHYSICAL EXAMINATION**												
Valdes et al[12] 2013 ¥	Not specified	Scaphoid shift test	Presence or absence of wrist pathology							2.76	0.25	The highly recommended test is only the scaphoid shift test. Reasonable tests are the midcarpal test.
		Midcarpal								2.67	Not calculated	
		Ulno-menisco-triquetral glide								1.2	0.77	
		Ballottement								1.12	0.83	
		Lunotriquetral test								1.03	0.80	
		GRIT								1.12	0.83	
		Ulnocarpal stress test								1.0	Unable to calculate	
Andersson et al[13] 2015	Not specified	Clinical tests	TFCC	1	0.58	0.69		0.71	0.55			Clinical provocation wrist tests were of limited diagnostic value.
			SLIL	1	0.61	0.79		0.68	0.74			
			LTIL	1	0.17	0.84		0.06	0.94			
**IMAGING**												
Hobby et al[10] 2000	Not specified	MRI	TFCC	11	0.83 (0.77 - 0.88)	0.80 (0.71 - 0.86)	0.81 (0.77-0.85)					High-resolution MRI was an accurate means of diagnosing TFCC tears; although MRI was highly specific for SL and LT ligament injuries, however, its sensitivity is low.
			SLIL	6	0.70 (0.50 - 0.82)	0.90 (0.57 - 0.99)	0.85 (0.66-0.94)					
			LTIL	6	0.56 (0.29 - 0.80)	0.91 (0.70 - 0.98)	0.82 (0.75-0.88)					
		MRI of high resolution	TFCC	3	0.88 (0.77 - 0.94)	0.88 (0.72 - 0.95)	0.88 (0.80-0.93)					
			SLIL	3	0.88 (0.68 - 0.96)	0.90 (0.57 - 0.99)	0.85 (0.66-0.94)					

For peer review only

For peer review only - http://bmjopen.bmj.com/site/about/guidelines.xhtml

			LTIL	3	0.75 (0.31 - 0.98)	0.90 (0.47 - 0.99)	0.86 (0.75-0.93)					
Andersson et al[13] 2015 ϕ	Not specified	MRI	TFCC	7	0.44-0.93	0.54-1.00		0.71 - 1.00	0.37 - 0.90			A negative result from MRI is unable to rule out the possibility of a clinically relevant injury to the TFCC, SL ligament, or LT ligament of the wrist.
			SLIL	7	0.11-0.89	0.55-1.00		0.25 - 1.00	0.72 - 0.94			
			LTIL	7	0.00-0.82	0.76-1.00		0.00 - 1.00	0.74 - 0.95			
Smith et al[14] 2012	Not specified	MRI	TFCC	17	0.75 (0.70 - 0.79)	0.81 (0.76 - 0.86)						MRA is recommended rather than MRI.
		MRA	TFCC	8	0.84 (0.79 - 0.89)	0.95 (0.92 - 0.98)						

MRI: Magnetic Resonance Imaging
MRA: Magnetic Resonance Arthrography
TFCC: Triangular fibrocartilage complex
SLIL: Scapholunate interosseous ligament
LTIL: Lunotriquetral interosseous ligament
GRIT: griping rotatory impaction test
ϕ Sensitivity, Specificity, Positive Predictive value and Negative Predictive value range described, because of the high heterogeneity.
¥ Positive and negative Likelihood ratio value range described, because of the high heterogeneity.

Within the available systematic reviews, published up to 2015, no studies were found on the diagnostic accuracy of history taking and only the scaphoid shift test and high-resolution MRI were recommended for diagnosing TFCC tears. [10 12-14]

The main purpose of the present study was to provide a systematic overview of the diagnostic accuracy of history taking, physical examination and imaging for detecting non-chronic ligament and tendon injuries of the finger, hand and wrist. The secondary aim of this study was to retrieve the clinical care setting (hospital or non-institutionalized GP) of the eligible studies and the studies published in previous systematic reviews.

Methods

The Preferred Reporting Items for Systematic Reviews and Meta-Analyses (PRISMA)

For peer review only

For peer review only - http://bmjopen.bmj.com/site/about/guidelines.xhtml

Statement was used to guide the conduct and reporting of the study.[15] A review protocol was composed prior to searching the literature, but central registration was not completed.

Search strategy

A Biomedical Information specialist (Wichor M. Bramer) from the Medical Library at Erasmus MC performed a search for studies in Medline, Embase, Cochrane Library, Web of Science, Google scholar ProQuest and Cinahl from 2000 up to 6 February 2019. This starting point was used since multiple reviews are available that already cover the period up to the year 2000 (Table 1). Search terms included hand, finger and wrist injuries, history taking, provocative test(s), diagnostic test(s) and imaging tests. The full electronic search strategy for the Embase database is presented in Appendix 1.

Study Selection criteria

The following selection criteria were used:

- The study investigated the diagnostic accuracy;
- history taking, physical examination or imaging as an index test;
- non-chronic hand, finger or wrist injuries;
- adult study population (minimum age ≥ 16 years);
- diagnostic accuracy was reported or could be calculated;
- case reports, reviews and conference proceedings were excluded;
- distal radius and ulna injuries were excluded.

Surgical observations (arthroscopy) are the reference standard for confirming a diagnosis of non-chronic hand, finger or wrist injury, although only a subset of patients suspected of having non-chronic hand, finger or wrist injury require surgery. To decrease verification bias, diagnostic-imaging techniques for non-chronic hand injury were accepted as reference tests as well. Since tendinopathy does not typically require surgery, imaging is also a pragmatic

For peer review only - http://bmjopen.bmj.com/site/about/guidelines.xhtml

reference standard for this condition. As this review focused on non-chronic pathologies, studies including patient with chronic pathologies (e.g. osteoarthritis, rheumatic arthritis) were excluded. Infection and neurologic injuries are out of the scope of this review and are therefore not included. Carpal tunnel syndrome is extensively described in the literature and was therefore not included in this review.[16-18] Diagnoses of musculoskeletal soft-tissue tumours were also excluded. No language restrictions were applied.

Two reviewers (PK, YA) read all titles and abstracts independently. Articles that could not be excluded on the basis of the title and/or abstract were retrieved in full text and were read and checked for inclusion by the two reviewers independently. If there was no agreement, a third reviewer (JR) made the final decision. In addition, the reference lists of all included studies were reviewed to check for additional relevant studies.

Data collection process and Methodological Quality Assessment

Two reviewers (PK, JR) independently extracted the data. Data were extracted describing the study design, characteristics of the study population, test characteristics, setting (hospital care or non-institutionalized GP care), and diagnostic parameters. The following values were extracted, when documented: sensitivity (Se), specificity (Sp), accuracy, positive predictive value (PPV), negative predictive value (NPV) and likelihood ratio (LR). If diagnostic parameters were not reported, they were calculated from reported data or authors were contacted by email when data was unavailable. If an included study presented results from multiple independent observers, accuracy measures were averaged over the observers. Furthermore, data of the studies published in previous systematic reviews were extracted describing the setting (hospital care or non-institutionalized GP care). Methodological quality was assessed using the Quality Assessment of Diagnostic Accuracy Studies (QUADAS-2) checklist.[19] This tool allows more transparent rating of bias and applicability in primary diagnostic accuracy studies. The QUADAS-2 tool consists of four domains: patient selection,

For peer review only - http://bmjopen.bmj.com/site/about/guidelines.xhtml

index test, reference standard, and flow and timing. Two reviewers (PK, JR) independently assessed the risk of bias and applicability of each included study. Disagreements were resolved by discussion. Questions were answered with ‘yes’, ‘no’ or ‘unclear’.

Patient and public involvement

Patients and members of the public were not involved in this systematic review update.

For peer review only

Results

Study Selection

The flow diagram for the categorization process is presented in Figure 1. We assessed 209 full-text articles for eligibility out of 4867 records identified through database searches. A total of 23 diagnostic studies were finally identified, assessed and interpreted.

Study characteristics

The characteristics of the studies are presented in Table 2.

Table 2. Characteristics of the Eligible Studies (N=23)

**Author, year**	**Participants**	**Design**	**Setting (Country)**	**Trauma**	**Index test 1**	**Index test 2**	**Reference test**
**Wrist injuries**							
Anderson[20] (2008)	102	Retrospective	Not described (USA)	TFCC/SLIL /LTIL/UTIL	MRI 1,5T	MRI 3T	Arthroscopy
Pahwa2[1] (2014)	53	Prospective	Not described (India)	TFCC/SLIL/LTIL	MRI 1,5T	MR arthrography	Arthroscopy

For peer review only - http://bmjopen.bmj.com/site/about/guidelines.xhtml

Prosser[22] (2011)	105	Prospective	Private hand clinic (Australia)	TFCC/SLIL/LTIL	MRI 1T	Provocative tests	Arthroscopy
Langner[23] (2015)	38	Not described	Not described (Germany)	SL dissociation	Cine MRI 3T	Cineradiography	Arthroscopy
Spaans[24] (2013)	37	Not described	Department for Hand and Plastic Surgery # (Netherlands)	SLIL (complete tear)	MRI 3T		Arthrotomy
Greditzer[28] (2016)	26	Retrospective	Department for Hand and Plastic Surgery# (USA)	SLIL	MRI 1,5T axial sequences	MRI 1,5T coronal sequences	Arthroscopy
Al-Hiari[25] (2013)	42	Prospective	Orthopedic Surgery# (Jordan)	TFCC (full-thickness tears)	MR arthrography		Arthroscopy
Schmauss[26] (2016)	908	Retrospective	Department for Hand and Plastic Surgery (Germany)	TFCC	MRI (resolution not described)	Provocative tests	Arthroscopy
Lee[27] (2016)	39	Prospective	Not described (China)	TFCC (full-thickness tears)/SLIL/LTIL	MR (3T) arthrography without traction	MR (3T) arthrography with traction	Conventional arthrography
Finlay[29] (2004)	26	Retrospective	Not described (Canada)	TFCC/SLIL/LTIL	US (9-13MHz)		MR arthrography**
Dornberger[30] (2015)	72	Prospective	Hand surgery# (Germany)	SLIL	Radiographs		Arthroscopy

For peer review only

Koskinen[31] (2012)	52	Not described	Not described (Finland)	TFCC/ SLIL/ LTIL	CBCT arthrography *		MR arthrography
Boer[32] (2018)	150	Retrospective	Plastic or Orthopedic Surgery (Netherlands)	TFCC	MRI 1,5 or 3,0T	MRA 1,5 or 3,0T	Arthroscopy
Lee[33] (2018)	65	Prospective	Emergency department (Korea)	TFCC	US		MRI 3,0T
Suojärvi[34] (2017)	21	Prospective	Hand surgery (Finland)	SLIL/LTIL/TFCC	CBCT arthrography	MRA	Arthroscopy
Mahmood[35] (2012)	30	Retrospective	General hospital (UK)	SLIL/LTIL/TFCC	MRA		Arthroscopy
**Hand and finger injuries**							
Lutsky[36] (2014)	20	Retrospective	Not described (USA)	Collateral ligament tears of the MPJ of the fingers	MRI (open,1,5T, 3T)		Surgical findings
Guntern[37] (2007)	8	Retrospective	Not described (Switzerland)	A2 pulley Lesion	Clinical examination		MRI 3T
Klauser[38] (2002)	64	Not described	Not described (Austria)	Finger pulley injuries	US (12MHz)		MRI 1,5T
Lee[39] (2000)	10	Not described	Not described (USA)	Flexor tendon injuries	US (L10-5MHz)		Surgical findings
Zhang[40] (2012)	92	Not described	Department of Surgery (China)	Flexor tendon injuries	US (10MHz)		Surgical findings
Mahajan[41] (2016)	30	Prospective	Emergency Room and outpatients clinic of Surgery and Orthopedics (Netherlands)	UCL injuries	Clinical examination		MRI 1,5T

For peer review only - http://bmjopen.bmj.com/site/about/guidelines.xhtml

Shekarchi[42] (2017)	20	Prospective	Emergency department (Iran)	UCL of the thumb	US		MRI

* Cone Beam CT arthrography

** Tricompartment wrist arthrography

\# setting for the study was obtained after email contact

TFCC: Triangular Fibrocartilage Complex
SLIL: Scapholunate Interosseous Ligament
UTIL: Ulnotriquetral Interosseous Ligament
LTIL: Lunotriquetral Ligament
UCL: Ulnar Collateral Ligament

Eight studies were retrospective[20 26 28 29 32 33 36 37], nine studies were prospective[21 22 25 27 30 33 34 42 43] and six studies[23 24 31 34-36] gave no description of the study design. Eight studies[20 22 26 30 32 33 38 39] included more than 60 participants; six of these studies[20 22 26 30 32 33] described wrist pathologies and two[38 39] described hand pathologies. In total, 16 studies[20-35] described injuries to the wrist anatomy and seven studies[36-42] described injuries to the hand/finger anatomy.

Quality Assessment

There was considerable underreporting of important quality domains in most studies (see Table 3).

Table 3. Summary of Methodological Quality According to Quality Assessment of Diagnostic Accuracy Studies-2.

**Author, year, index test(s)**	**Risk of Bias**				**Applicability Concerns**		
	Patient Selection	Index Test	Reference standard	Flow and Timing	Patient Selection	Index Test	Reference standard
**Wrist disabilities**							
Anderson[20] (2008)	LR	LR	HR	LR	LR	LR	LR
Pahwa[21] (2014)	UR	LR	HR	HR	LR	LR	LR
Prosser[22] (2011) Provocative tests	LR	LR	LR	LR	LR	LR	LR
MRI	LR	LR	HR	LR	LR	LR	LR
Langner[23] (2015)	LR	LR	HR	HR	LR	LR	LR

For peer review only - http://bmjopen.bmj.com/site/about/guidelines.xhtml

For peer review only

Spaans[24] (2013)	UR	LR	LR	UR	LR	LR	LR
Al-Hiari[25] (2013)	LR	LR	HR	LR	LR	LR	LR
Schmauss[26] (2016)	LR	HR	HR	LR	LR	LR	LR
Lee[27] (2016)	LR	HR	HR	LR	LR	LR	LR
Greditzer[28] (2016)	HR	LR	HR	LR	LR	LR	LR
Finlay[29] (2004)	UR	LR	LR	LR	LR	LR	LR
Dornberger[30] (2015)	LR	LR	HR	LR	LR	LR	LR
Koskinen[31] (2012)	LR	HR	HR	LR	LR	LR	LR
Boer[32] (2018)	HR	UR	HR	LR	LR	LR	LR
Lee[33] (2018)	LR	LR	LR	LR	LR	LR	LR
Suojärvi[34] (2017)	LR	LR	HR	HR	LR	LR	LR
Mahmood[35] (2012)	UR	LR	UR	LR	LR	LR	LR
**Hand and finger disabilities**							
Lutsky[36] (2014)	LR	UR	HR	LR	LR	LR	LR
Guntern[37] (2007)	LR	HR	LR	LR	LR	LR	LR
Klauser[38] (2002)	LR	HR	LR	HR	LR	LR	LR
Lee[39] (2000)	UR	LR	LR	LR	LR	LR	LR
Zhang[40] (2012)	LR	LR	HR	LR	LR	LR	LR
Mahajan[41] (2016)	LR	LR	LR	LR	LR	LR	LR
Shekarchi[42] (2017)	UR	LR	LR	LR	LR	LR	LR
Abbreviations:	LR: Low Risk	HR: High Risk	U: Unclear Risk				

Two studies had low risk of bias on all quality domains.[22 33] In eight[21 24 28 29 32 35 39 42] of the 23 studies, patient selection was not well documented. Furthermore, the risk of bias was predominantly influenced by the lack of a proper description of the index test (30%, 7/23)[26 27 31 32 36-38] or the reference standard (65%, 15/23)[20-23 25-28 30-32 34-36 40]. Regarding flow and timing, not all patients received the reference standard in four studies (22%, 5/23).[21 23 25 34 38] Due to our selection procedure, all the studies match the review question.

Accuracy of diagnostic tests concerning Wrist injuries

None of the studies evaluated the diagnostic accuracy of history taking. Physical examination was evaluated in 2 studies for diagnosing lesions of the triangular fibrocartilage complex

For peer review only - http://bmjopen.bmj.com/site/about/guidelines.xhtml

For peer review only

(TFCC).[22 26] Provocative wrist tests for diagnosing scapholunate interosseous ligament (SLIL) and lunotriquetral interosseous ligament (LTIL) lesions was assessed in one study.[22] Radiographs were used as an index test in 1 study for diagnosing SLIL lesions.[30] US for diagnosing TFCC lesions was used in 2 studies.[29 33] Two studies used Cone Beam Computed Tomography (CBCT) as index for diagnosing TFCC lesions.[31 34] In 12 studies MRI was used as an index test.[20-28 32 34 35] The accuracy of MRI for TFCC, SLIL, LTIL and UTIL lesions with high-resolution (3 tesla) techniques ranging from 89-91%, 75-92%, 91% and 100%, respectively. The accuracy of MRI for TFCC, SLIL, LTIL and UTIL lesions with low-resolution (1.5 tesla) techniques ranging from 81-100%, 67-81%, 81-94% and 95%, respectively. The accuracy measures of the diagnostic tests are presented in table 4.

Table 4. Accuracy of the Diagnostic Tests of the Wrist

**Author, year**	**Index test 1**	**Reference test**	**Trauma**	**Se %**	**Sp %**	**Accuracy % (95% CI)**	**PPV**	**NPV**	**LR +**	**LR -**
**PHYSICAL EXAMINATION**										
Prosser[22] (2011)	Provocative tests	Arthroscopy	TFCC	58	69	73	71	55	1.88 (1.15-3.04)	0.53 (0.33-0.86)
			SLIL	61	79	78	68	74	2.88 (1.68-4.92)	0.28 (0.15-0.55)
			LTIL	17	84	95	6	94	1.03 (.16-	0.80 (0.35-1.8)

For peer review only

For peer review only - http://bmjopen.bmj.com/site/about/guidelines.xhtml

									6.5 2)	
Schmauss[26] (2016)	Fovea sign	Arthroscopy	TFCC	73	44	58	53	66		
	Ulna grinding test			90	20	56	54	65		
**IMAGING: RADIOGRAPHS**										
Dornberger[30] (2015)	Radiographs (Stecher's projection)	Arthroscopy	SLIL	(76.9+80.8)/2**	(86.4+84.1)/2**	(92.7+90.6)/2**	(76.9+75)/2**	(86.4+88.1)/2**		
**IMAGING: US**										
Finlay[29] (2004)	US (9-13MHz)	MR arthrography Tricompartment	SLIL	100	100	100	100	100		
			TFCC	64	100	85	100	79		
			LTIL	25	100	77	100	75		
Lee[33] (2018)	US	MRI	TFCC, total	99**	88**	97**	97**	95**		
**IMAGING: MRI**										
Anderson[20] (2008)	MRI 1,5T	Arthroscopy	TFCC	82	59	83 (72.4-89.9)*				
			SLIL	57	83	78 (67.2-86.3)*				
			UTIL	57	89	95 (86.1-98.3)*				
			LTIL	22	94	86 (75.3-91.9)*				
	MRI 3T		TFCC	90	74	91 (75.8-96.8)*				
			SLIL	70	94	91 (75.8-96.8)*				

For peer review only

			UTIL	67	87	100 (97.9-100)*				
			LTIL	50	94	91 (75.8-96.8)*				
Pahwa[21] (2014)	MR arthrography	Arthroscopy	TFCC	100	100	100	100	100		
			SLIL	100	100	100	100	100		
			LTIL	100	100	100	100	100		
	MRI 1,5T MEDIC		TFCC	83	100	81	91	60		
			SLIL	63	100	81	100	73		
			LTIL	40	100	81	100	73		
	MRI FS PD/T2		TFCC	75	100	75	90	50		
			SLIL	38	100	69	100	62		
			LTIL	20	100	75	100	73		
Prosser[22] (2011)	MRI 1T	Arthroscopy	TFCC			86 (PT+MRI)			5.56 (1.92-16.1)	0.15 (0.06-0.37)
			SLIL			80 (PT+MRI)			4.17 (1.54-11.3)	0.32 (0.16-0.65)
			LTIL			94 (PT+MRI)			n/a	1.06 (0.99-1.14)

For peer review only

Schmauss[26] (2016)	MRI resolution not described	Arthroscopy		76	41	58	55	65		
Langner[23] (2015)	Cine MRI 3,0T and Cineradiography	Arthroscopy	SL dissociation	85	90	92			8.5	0.16
Spaans[24] (2013)	MRI 3T	Arthrotomy	SLIL	75.5**	100*	75**	98.5**	8*		
Greditzer[28] (2016)	MRI 1,5T axial sequences	Arthroscopy	SLIL	79	82	80	76	84		
	MRI 1,5T coronal sequences		SLIL	65	69	67	68	71		
Al-Hiari[25] (2013)	MR arthrography	Arthroscopy	TFCC	93	80	85				
Lee[27] (2016)	MR arthrography without traction	Conventional arthrography	TFCC	83	81	83	87	76		
			SLIL	66	97	95	67	97		
			LTIL	57	94	88	67	91		
	MR arthrography with traction		TFCC	96	100	98	100	94		
			SLIL	100	100	100	100	100		
			LTIL	100	100	100	100	100		
Boer[32] (2018)	MRI 1,5T	Arthroscopy	TFCC	71	75	100	71	75		
	MRI 3,0T	Arthroscopy	TFCC	73	67	89	83	52		

For peer review only

For peer review only - http://bmjopen.bmj.com/site/about/guidelines.xhtml

	MR arthrography 1,5T	Arthroscopy	TFCC	80	100	80	100	50		
	MR arthrography 3,0T	Arthroscopy	TFCC	73	100	73	100	60		
Suojärvi [34] (2017)	MR arthrography	Arthroscopy	SLIL	25 (3-65)	80 (61-92)	68 (51-83)	25 (3-65)	80 (61-92)		
			LTIL	50 (7-93)	77 (59-90)	74 (57-88)	22 (3-60)	92 (75-99)		
			TFCC	44 (22-69)	50 (25-75)	47 (30-65)	50 (25-75)	44 (21-69)		
			SLIL or LTIL	33 (7-60)	79 (67-88)	72 (56-82)	24 (7-50)	86 (74-94)		
Mahmood [35] (2012)	MR arthrography	Arthroscopy	SLIL	91	88		83	88		
			LTIL	100	100		100	100		
			TFCC	90	75		85	80		
**IMAGING: COMPUTED TOMOGRAPHY**										
Koskinen [31] (2012)	CBCT arthrography	MR arthrography	TFCC	76	90	87	83	87		
			SLIL	56	91	83	67	89		
			LTIL	83	81	82	44	96		
Suojärvi [34] (2017)	CBCT	Arthroscopy	SLIL	63 (24-91)	87 (69-96)	82 (66-92)	56 921-86)	90 (73-98)		
			LTIL	100 (40-100)	59 (41-76)	64 (46-79)	24 (7-50)	100 (83-100)		
			TFCC	67 (40-87)	89 (63-98)	77 (60-90)	86 (57-98)	73 (50-89)		
			SLIL or LTIL	75 (43-95)	76 (65-86)	73 (61-83)	35 (16-53)	95 (86-99)		

TFCC: Triangular fibrocartilage complex

SLIL: Scapholunate interosseous ligament

UTIL: Ulnotriquetral interosseous ligament

LTIL: Lunotriquetral ligament

For peer review only

CBCT:	Cone Beam Computed Tomography
MRI:	Magnetic resonance imaging
T:	Tesla
US:	Ultra Sonography
Se:	Sensitivity
Sp:	Specificity
PPV:	Positive predictive value
NPV:	Negative predictive value
LR:	Likelihood ratio
n/a:	not available due to low prevalence
*	Only reported for one of two readers.
**	Average between presented individual values of two readers.
***	SL distance 3.7mm on Stecher's projection

In addition to the data presented in Table 4, the study of Schmauss et al. presented the diagnostic accuracy of their tests separately for different subgroups.[26] These results are summarized in Appendix 2.

Accuracy of diagnostic tests concerning Hand and Finger injuries

Table 5 describes the accuracy of the diagnostic test for non-chronic hand and fingers injuries.[36-42]

Table 5. Accuracy of the Diagnostic Tests of the Hand and Fingers.

**Author, year**	**Index test 1**	**Reference test**	**Trauma**	**Se (%)**	**Sp (%)**	**Accuracy (%)**	**PPV (%)**	**NPV (%)**
Lutsky[36] (2014)	MRI (open,1,5Tor 3T)	Surgical findings	Collateral ligament tears of the MPJ of the fingers	64	∞	64	100	∞
Guntern[37] (2007)	Clinical examination	MRI 3T	A2 pulley Lesion	88	100	88	100	95
Klauser[38] (2002)	US (12MHz)	MRI 1,5T (and Surgical findings, n=7)	Finger pulley injuries	98	100	99	100	97

For peer review only - http://bmjopen.bmj.com/site/about/guidelines.xhtml

Lee[39] (2000)	US (10-5MHz)	Surgical findings	Flexor tendon injuries			90		
Zhang[40] (2012)	US (10MHz)	Surgical findings	Flexor tendon injuries			100		
	History and Clinical examination					34		
Mahajan[41] (2016)	Clinical examination	MRI 1,5T	UCL injuries	91	75	87	91	75
Shekarchi[42] (2017)	US	MRI	UCL of the thumb	71 (30-95)	85 (54-97)	80	71 (30-95)	85 (54-97)

MRI: Magnetic resonance imaging
T: Tesla
US: Ultra Sonography
UCL: Ulnar Collateral Ligament
Se: Sensitivity
Sp: Specificity
PPV: Positive predictive value
NPV: Negative predictive value
LR: Likelihood ratio
∞: Infinity

For peer review only

Two studies concerned flexor tendon injuries[39 40], while the other studies concerned collateral ligament tears of the metacarpophalangeal Joint (MPJ) of the fingers[36], A2 pulley lesions[37], finger pulley injuries[38] and ulnar collateral ligament injuries[41 42]. None of the studies involved history taking. Clinical examination was used three times as an index test.[37 40 41] The Se, Sp, accuracy, PPV and NPV of physical examination in hand and finger injuries ranged from 88 to 99%, 75 to 100%, 34 to 88%, 91 to 100% and 75 to 95%, respectively. MRI was used once as an index test.[36] Four studies used ultrasonography (US) as an index test.[38-40 42] The accuracy of US in flexor injuries ranged from 90 to 100%.[39 40] The accuracy of US for finger pulley injuries and Ulnar Collateral Ligament (UCL) of the thumb was 99% and 80%, respectively.[38 42]

For peer review only - http://bmjopen.bmj.com/site/about/guidelines.xhtml

Clinical care setting

The clinical care setting was described in 9 out of 23 studies and was obtained by contacting the authors for an additional 4 studies: a private hand clinic[22], emergency department[33 42], department for hand and plastic surgery[24 26 28 30 32], surgery[40], orthopaedics department[25 32] and in an emergency room and outpatient clinic of a surgery and orthopaedics department[41]. Despite multiple attempts to contact the authors by email, clarification regarding the setting could not be obtained for the remaining 10 studies.

Discussion

The standard diagnostic work-up for non-chronic finger, hand and wrist trauma consists of history taking, a physical examination and, if needed, imaging. There is general agreement that a detailed patient history and a conscientious clinical examination should be standard methods of diagnosing wrist pain.[9] The main purpose of the present study was to systematically examine the diagnostic accuracy of history taking, physical examination and imaging for detecting non-chronic ligament and tendon injuries of the finger, hand and wrist.

Diagnostic Accuracy of the Diagnostic Tests of the Wrist

Although common practice in hospital care, in previous reviews[10 12-14] and current systematic review update, no studies were identified on the diagnostic accuracy of history taking for non-chronic ligament and tendon injuries of the wrist.

This systematic review update included one new study on physical examinations for diagnosing non-chronic ligament and tendon injuries of the wrist, which did not affect the previous conclusion that physical examination is of limited value for diagnosing non-chronic ligament and tendon injuries of the wrist.[26]

For peer review only - http://bmjopen.bmj.com/site/about/guidelines.xhtml

In previous reviews, only the diagnostic performance for MRI and/or MR Arthrography (MRA) of the wrist were examined. This showed that high-resolution MRI was an accurate means for diagnosing TFCC tears and MRI was slightly specific for tears of the intrinsic ligament, but its sensitivity is low.[10 13] Beside MRA, rather than MRI, was recommended to be used in daily practise for the diagnosis of TFCC injuries.[10 37] In current review, the accuracy measures for MRI showed a wide range in diagnostic outcome values, with diagnostic accuracy measures no better for imaging at 3 tesla than at 1.5 tesla. As previously shown for full-thickness TFCC injuries, the present results indicate that the accuracy for tears of the TFCC, SLIL and LTIL is increased by MRA.[14] Computed tomography (CT) arthrography is an alternative in patients when MRI is contraindicated or when MRI is not available.[31]

In current review, 5 studies used another imaging tool, namely radiograph[30], US[29 33] and CBCT[31 34] for diagnosing non-chronic ligament and tendon injuries of the wrist. The diagnostic accuracy of radiograph was limited. Examination of SLIL and TFCC with US showed promising results and the added value should be further explored. Based on the included studies, CBCT has no added value in assessing non-chronic ligament and tendon injuries of the wrist, especially when we take the methodological quality of the studies into account.

However, a dynamic 4D-CT for the detection of SLIL or LTIL injuries is promising.[44 45] Nevertheless, the diagnostic accuracy has not yet been studied. At present, there is still insufficient scientific evidence regarding the ideal imaging technique for non-chronic intrinsic carpal ligament injuries of the wrist.

In current systematic review update and previous systematic reviews, the reported diagnostic accuracy measures for imaging modalities were characterized by markedly heterogeneous results. It was not appropriate to pool results for the diagnostic accuracy of imaging, due to a

For peer review only - http://bmjopen.bmj.com/site/about/guidelines.xhtml

lack of multiple imaging studies on one specific wrist injury. Based on previews and current review we can conclude that MRA rather than MRI is the preferred imaging tool in hospital care setting for detecting non-chronic ligament and tendon injuries of the wrist.

Diagnostic Accuracy of the Diagnostic Tests of the Hand and the Fingers

According to our knowledge, there are no reviews previously published to date on the diagnostic accuracy of history taking, physical examination and imaging for non-chronic ligament and tendon injuries of the finger and hand.

We identified 3 studies on the diagnostic accuracy of history taking and/or clinical examination.[37 40 41] One study[41] had no methodological limitation, while the other two studies had methodological flaws (high risk of bias) on index test[37] and reference standard[40]. Beside, each study evaluated different diagnostics tests for different pathologies. So there is limited evidence on the diagnostic accuracy of history taking and physical examination for diagnosing hand and finger injuries.

Imaging studies exhibit a wide variety of imaging tools and studied pathologies. Moreover, studies with imaging tools as a diagnostic modality had methodological flaws and serious limitations, so we have to interpret these results with caution. Only the study of Lee et al. had relatively few methodological flaws.[39] These authors showed that ultrasonography can possibly help to evaluate completely lacerated flexor tendon injuries. Nevertheless, as indicated by the authors, ultrasonography cannot accurately determine the status of partially transected tendons.[39]

Clinical care setting

The secondary aim of this study was to retrieve the clinical care setting (hospital or non-institutionalized GP) of the eligible studies and the studies published in previous systematic

For peer review only - http://bmjopen.bmj.com/site/about/guidelines.xhtml

reviews. We assume that all studies included in the current and previous reviews were done in a hospital care setting; this was either described in the paper, was confirmed by the authors, or due to the fact that all authors of the remaining studies were only affiliated to hospitals.

It is essential to distinguish between diagnosing these injuries in hospital care and in non-institutionalized GP care, as results from diagnostic studies in hospital care cannot automatically be translated into guidelines for non-institutionalized GP care.[11] Since previous systematic reviews and the current update of the literature did not identify any studies performed in non-institutionalized GP care, it is not possible to advise GPs conscientiously. Given the burden of non-chronic hand and wrist trauma in non-institutionalized GP care, diagnostic studies focusing on non-chronic hand, finger and wrist ligament and tendon injuries are urgently needed.[1 2]

Conclusions:

Our systematic review showed that the gap in knowledge to provide evidence for diagnostic tests to diagnose non-chronic finger, hand and wrist ligament and tendon injuries remains challenging. Based on previous reviews and our systematic review update we had to conclude that physical examination is of limited value for diagnosing non-chronic ligament and tendon injuries of the wrist. Further, MRA rather than MRI is the preferred imaging tool for detecting non-chronic ligament and tendon injuries of the wrist in hospital care setting, while the evidence is lacking on the preferred imaging tool for hand and/or finger injuries and for other clinical settings.

Abbreviations

CBCT: Cone Beam Computer Tomography

For peer review only - http://bmjopen.bmj.com/site/about/guidelines.xhtml

GRIT: Griping rotatory impaction test

HR: High Risk (QUADAS-2)

LR: Likelihood ratio

LR: Low Risk (QUADAS-2)

LTIL: Lunotriquetral interosseous ligament

MRA: Magnetic Resonance Arthrography

MRI: Magnetic Resonance Imaging

NPV: Negative predictive value

PPV: Positive predictive value

Se: Sensitivity

Sp: Specificity

SLIL: Scapholunate interosseous ligament

T: Tesla

TFCC: Triangular fibrocartilage complex

UCL: Ulnar Collateral Ligament

UR: Unclear Risk (QUADAS-2)

US: Ultra Sonography

UTIL: Ulnotriquetral Interosseous Ligament

Acknowledgement

The authors thank Wichor Bramer (Biomedical information specialist of Erasmus University Medical Center Rotterdam Medical Library) for help with the electronic search strategies and Yassine Aaboubout (MSc) for helping with study selection and extracting the data.

Authors' contributions

For peer review only

PK, NM, SB, GK and JR all contributed to the design of the study. PK and JR were responsible for article selection and analysed the data. All authors contributed to writing and revision of the manuscript. All authors have given approval of the submitted version of the manuscript and agree to be accountable for all aspects of the work.

Funding

No funding.

Availability of data and materials

The datasets used and/or analysed during the current study are available from the corresponding author on reasonable request.

Ethics approval and consent to participate

Not applicable.

Consent for publication

Not applicable.

Competing interests

The authors declare that they have no competing interests.

For peer review only

For peer review only - http://bmjopen.bmj.com/site/about/guidelines.xhtml

REFERENCES

1. Owen RA, Melton III LJ, Johnson KA, Ilstrup DM, Riggs BL. Incidence of Colles' fracture in a North American community. Am J Public Health 1982;72:605–7.
2. McCullough NP, Smith FW, Cooper JG. Early MRI in the management of the clinical scaphoid fracture. Eur J Emerg Med. 2011;18(3):133–6.
3. Larsen CF, Mulder S, Johansen AM, Stam C. The epidemiology of hand injuries in The Netherlands and Denmark. Eur J Epidemiol. 2004;19(4):323-7.
4. MW van der Linden, GP Westert, DH de Bakker, FG Schellevis. Tweede Nationale Studie naar ziekten en verrichtingen in de huisartspraktijk. Klachten en aandoeningen in de bevolking en in de huisartspraktijk. Utrecht/Bilthoven: NIVEL/RIVM, 2004.
5. de Putter CE, Selles RW, Polinder S, Panneman MJ, Hovius SE, van Beeck EF. Economic impact of hand and wrist injuries: health-care costs and productivity costs in a population-based study. J Bone Joint Surg Am. 2012 May 2; 94(9).
6. Rosberg HE, Carlsson KS, Ceder-lund RI, Ramel E, Dahlin LB. Costs and outcome for serious hand and arm injuries during the first year after trauma – a prospective study. BMC Public Health 2013; 13:501.
7. Sahin F, Akca H, Akkaya N,Zincir OD, Isik A. Cost analysis and related factors in patients with traumatic hand injury. J. Hand Surg. Eur Vol 2013; 38: 673–9.
8. Schaub TA, Chung KC. Systems of provision and delivery of hand care, and its impact on the community. Injury. 2006 Nov;37(11):1066-70.
9. Taleisnik J. Clinical and technologic evaluation of ulnar wrist pain. J Hand Surg [Am] 13:801 - 802,1988.
10. Hobby JL, Tom BD, Bearcroft PW, Dixon AK. Magnetic resonance imaging of the wrist: diagnostic performance statistics. Clin Radiol. 2001 Jan; 56(1):50-7.
11. Nicholas Steel, Asmaa Abdelhamid, Tim Stokes, Helen Edwards, Robert Fleetcroft,

For peer review only

Amanda Howe, and Nadeem Qureshi. A review of clinical practice guidelines found that they were often based on evidence of uncertain relevance to primary care patients. J Clin Epidemiol. 2014 Nov; 67(11):1251–1257.

12. Valdes K, La Stayo PJ. The value of provocative tests for the wrist and elbow: a literature review. Hand Ther. 2013 Jan-Mar; 26(1): 32-42.
13. Andersson JK, Andernord D, Karlsson J, Fridén J. Efficacy of Magnetic Resonance Imaging and Clinical Tests in Diagnostics of Wrist Ligament Injuries: A Systematic Review. Arthroscopy. 2015 Oct; 31(10):2014-20.
14. Smith TO, Drew B, Toms AP, Jerosch-Herold C, Chojnowski AJ. Diagnostic accuracy of magnetic resonance imaging and magnetic resonance arthrography for triangular fibrocartilaginous complex injury: a systematic review and meta-analysis. J Bone Joint Surg Am. 2012 May 2;94(9):824-32.
15. Moher D, Liberati A, Tetzlaff J, Altman DG; PRISMA group. Preferred reporting items for systematic reviews and meta-analyses: the PRISMA statement. BMJ. 2009; 339: b2535.
16. Gillig JD, White SD, Rachel JN. Acute Carpal Tunnel Syndrome: A Review of Current Literature.
Orthop Clin North Am. 2016 Jul;47(3):599-607.
17. Thawait GK, Subhawong TK, Thawait SK, Andreisek G, Belzberg AJ, Eng J, Carrino JA, Chhabra A. Magnetic resonance neurography of median neuropathies proximal to the carpal tunnel. Skeletal Radiol. 2012 Jun;41(6):623-32.
18. Duckworth AD, Jenkins PJ, McEachan JE. Diagnosing carpal tunnel syndrome. J Hand Surg Am. 2014 Jul;39(7):1403-7.
19. Whiting PF, Rutjes AW, Westwood ME, Mallett S, Deeks JJ, Reitsma JB, Leeflang MM, Sterne JA, Bossuyt PM. QUADAS-2: a revised tool for the quality assessment of

For peer review only - http://bmjopen.bmj.com/site/about/guidelines.xhtml

diagnostic accuracy studies. QUADAS-2 Group. Ann Intern Med. 2011 Oct 18; 155(8):529-36.

20. Anderson ML, Skinner JA, Felmlee JP, Berger RA, Amrami KK. Diagnostic comparison of 1.5 Tesla and 3.0 Tesla preoperative MRI of the wrist in patients with ulnar-sided wrist pain. J Hand Surg Am. 2008 Sep;33(7):1153-9.

21. Pahwa S, Srivastava DN, Sharma R, Gamanagatti S, Kotwal PP, Sharma V. Comparison of conventional MRI and MR arthrography in the evaluation wrist ligament tears: A preliminary experience. Indian J Radiol Imaging. 2014 Jul; 24(3):259-67.

22. Prosser R, Harvey L, La Stayo P, Hargreaves I, Scougall P, Herbert RD. Provocative wrist tests and MRI are of limited diagnostic value for suspected wrist ligament injuries: a cross-sectional study. J Physiother. 2011; 57(4):247-53.

23. Langner I, Fischer S, Eisenschenk A, Langner S. Cine MRI: a new approach to the diagnosis of scapholunate dissociation. Skeletal Radiol. 2015 Aug; 44(8): 1103-10.

24. Spaans AJ, Minnen Pv, Prins HJ, Korteweg MA, Schuurman AH. The value of 3.0-tesla MRI in diagnosing scapholunate ligament injury. J Wrist Surg. 2013 Feb;2(1): 69-72.

25. Al-Hiari AA. The role of wrist magnetic resonance arthrography in diagnosing triangular fibrocartilage complex tears: experience at king hussein medical center, jordan. Sultan Qaboos Univ Med J. 2013 May; 13(2):280-6.

26. Schmauss D, Pöhlmann S, Lohmeyer JA, Germann G, Bickert B, Megerle K. Clinical tests and magnetic resonance imaging have limited diagnostic value for triangular fibrocartilaginous complex lesions. Arch Orthop Trauma Surg. 2016 Jun;136(6):873-80.

27. Lee RK, Griffith JF, Ng AW, Nung RC, Yeung DK. Wrist Traction During MR Arthrography Improves Detection of Triangular Fibrocartilage Complex and Intrinsic Ligament Tears and Visibility of Articular Cartilage. AJR Am J Roentgenol. 2016

For peer review only

For peer review only - http://bmjopen.bmj.com/site/about/guidelines.xhtml

Jan;206(1):155-61.

28. Greditzer HG 4th, Zeidenberg J, Kam CC, Gray RR, Clifford PD, Mintz DN, Jose J. Optimal detection of scapholunate ligament tears with MRI. Acta Radiol. 2016 Feb 9.
29. Finlay K, Lee R, Friedman L. Ultrasound of intrinsic wrist ligament and triangular fibrocartilage injuries. Skeletal Radiol. 2004 Feb; 33(2): 85-90.
30. Dornberger JE, Rademacher G, Mutze S, Eisenschenk A, Stengel D. Accuracy of simple plain radiographic signs and measures to diagnose acute scapholunate ligament injuries of the wrist. Eur Radiol. 2015 Dec;25(12): 3488-98.
31. Koskinen SK, Haapamäki VV, Salo J, Lindfors NC, Kortesniemi M, Seppälä L, Mattila KT. CT arthrography of the wrist using a novel, mobile, dedicated extremity cone-beam CT (CBCT). Skeletal Radiol. 2013 May;42(5): 649-57.
32. Boer BC, Vestering M, van Raak SM, van Kooten EO, Huis In 't Veld R, Vochteloo AJH. MR arthrography is slightly more accurate than conventional MRI in detecting TFCC lesions of the wrist. Eur J Orthop Surg Traumatol. 2018 Dec;28(8):1549-1553.
33. Lee SH, Yun SJ. Point-of-care wrist ultrasonography in trauma patients with ulnar-sided pain and instability. Am J Emerg Med. 2018 May;36(5):859-864.
34. Suojärvi N, Haapamäki V, Lindfors N, Koskinen SK. Radiocarpal Injuries: Cone Beam Computed Tomography Arthrography, Magnetic Resonance Arthrography, and Arthroscopic Correlation among 21 Patients. Scand J Surg. 2017 Jun;106(2):173-179.
35. Mahmood A, Fountain J, Vasireddy N, Waseem M. Wrist MRI Arthrogram v Wrist Arthroscopy: What are we Finding? Open Orthop J. 2012;6:194-8.
36. Lutsky K, Levi D, Beredjiklian P. Utility of MRI for diagnosing complete tears of the collateral ligaments of the metacarpophalangeal joints of the lesser digits. Hand (N Y). 2014 Mar;9(1): 112-6.
37. Guntern D, Goncalves-Matoso V, Gray A, Picht C, Schnyder P, Theumann N. Finger

For peer review only - http://bmjopen.bmj.com/site/about/guidelines.xhtml

A2 pulley lesions in rock climbers: detection and characterization with magnetic resonance imaging at 3 Tesla--initial results. Invest Radiol. 2007 Jun; 42(6):435-41.

38. Klauser A, Frauscher F, Bodner G, Halpern EJ, Schocke MF, Springer P, Gabl M, Judmaier W, zur Nedden D. Finger pulley injuries in extreme rock climbers: depiction with dynamic US. Radiology. 2002 Mar; 222(3):755-61.

39. Lee DH, Robbin ML, Galliott R, Graveman VA. Ultrasound evaluation of flexor tendon lacerations. J Hand Surg Am. 2000 Mar;25(2):236-41.

40. Zhang GY, Zhuang HY, Wang LX. Value of high frequency ultrasonography in diagnosis and surgical repair of traumatic finger tendon ruptures. Med Princ Pract. 2012; 21(5):472-5.

41. Mahajan M, Tolman C, Würth B, Rhemrev SJ. Clinical evaluation vs magnetic resonance imaging of the skier's thumb: A prospective cohort of 30 patients. Eur J Radiol. 2016 Oct;85(10):1750-6.

42. Shekarchi B, Mokhdanzadeh Dashti M, Shahrezaei M, Karimi E. The Accuracy of Ultrasonography in Detection of Ulnar Collateral Ligament of Thumb Injuries; a Cross-Sectional Study. Emerg (Tehran). 2018;6(1):e15.

43. Whiting P, Rutjes AW, Reitsma JB, Glas AS, Bossuyt PM, Kleijnen J. Sources of variation and bias in studies of diagnostic accuracy: a systematic review. Ann Intern Med. 2004 Feb 3;140(3):189-202. Review.

44. Kakar S, Breighner RE, Leng S, McCollough CH, Moran SL, Berger RA, Zhao KD. The Role of Dynamic (4D) CT in the Detection of Scapholunate Ligament Injury. J Wrist Surg. 2016 Nov;5(4):306-310.

45. Zhao K, Breighner R, Holmes III D, Leng S, McCollough C, An K. J Biomech Eng. A Technique for Quantifying Wrist Motion Using Four-Dimensional Computed Tomography: Approach and Validation**.** 2015 Jul;137(7).

For peer review only - http://bmjopen.bmj.com/site/about/guidelines.xhtml

Figure 1. Flow chart study selection

For peer review only

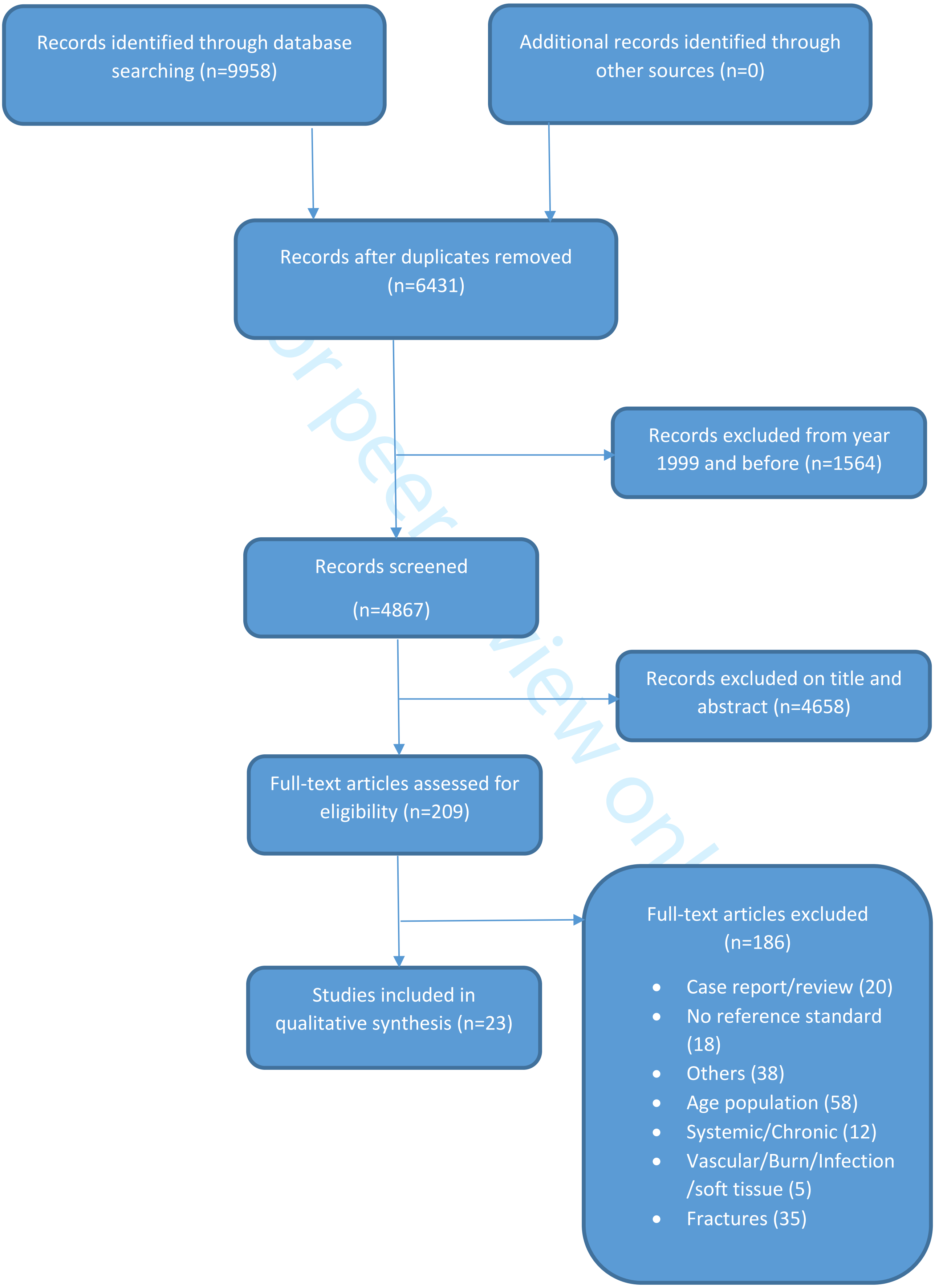
Records identified through database searching (n=9958)
Additional records identified through other sources (n=0)
Records after duplicates removed (n=6431)
Records excluded from year 1999 and before (n=1564)
Records screened
(n=4867)
Records excluded on title and abstract (n=4658)
Full-text articles assessed for eligibility (n=209)
Full-text articles excluded (n=186)
Case report/review (20)
No reference standard (18)
Others (38)
Age population (58)
Systemic/Chronic (12)
Vascular/Burn/Infection /soft tissue (5)
Fractures (35)
Studies included in qualitative synthesis (n=23)

Appendix 1

Database	Search terms
Embase	('hand injury'/exp OR 'wrist injury'/exp OR 'wrist fracture'/exp OR (('hand bone'/exp OR wrist/exp OR hand/exp OR 'wrist pain'/exp OR 'hand pain'/exp) AND ('bone injury'/exp OR fracture/de OR 'ligament injury'/exp OR 'ligament rupture'/exp)) OR (((hand OR hands OR wrist* OR finger* OR carpal* OR carpus OR phalanx* OR metacarp* OR capitate* OR hamat* OR lunat* OR pisiform* OR scaphoid* OR trapezium* OR trapezoid* OR triquetr* OR navicular* OR lunar OR semilunar* OR multangulum* OR pyramid* OR metacarpophalang* OR thumb* OR 'distal radius' OR 'distal ulna' OR 'distal radial' OR 'distal ulnar' OR scapholunate* OR lunotriquetral* OR 'triangular fibrocartilaginous' OR SLIL OR LTIL OR tfcc OR 'ulnar collateral ligament' OR 'ulnar collateral ligaments' OR ucl) NEAR/3 (injur* OR trauma* OR wound* OR lesion* OR dislocate* OR fracture* OR damage* OR tear* OR sprain* OR displace* OR rupture*))):ab,ti) AND ('diagnostic test'/de OR 'function test'/exp OR 'diagnostic error'/exp OR 'diagnostic accuracy'/exp OR 'diagnostic value'/exp OR 'differential diagnosis'/exp OR 'delayed diagnosis'/exp OR 'sensitivity and specificity'/exp OR (((diagnos* OR detect* OR differen* OR strength* OR motion*) NEAR/3 (test* OR accura* OR error* OR false OR fail* OR value* OR impact* OR effective* OR earl* OR missed OR correct* OR incorrect* OR delay* OR difficult* OR negative* OR positive* OR sensitivit* OR specificit* OR confirm* OR abilit*)) OR (diagnos* NEAR/3 differen*) OR misdiagnos* OR underdiagnos* OR undetect* OR (predict* NEAR/3 value*) OR (function* NEAR/3 test*) OR (false NEAR/3 (negative* OR positive*))):ab,ti) NOT ([Conference Abstract]/lim OR [Letter]/lim OR [Note]/lim OR [Editorial]/lim) AND [english]/lim NOT ([animals]/lim NOT [humans]/lim)

Search terms for the other databases are available upon request.

For peer review only

Appendix 2. Diagnostic Accuracy of Tests in Different Subgroups Presented in the Study of Schmauss et al.[26]

	**Se (%)**	**Sp (%)**	**Accuracy (%)**	**PPV (%)**	**NPV (%)**
Ulna carpal pain					
Ulnar fovea sign	88	9	54	56	39
Ulnar grinding test	93	12	58	58	58
MRI	88	23	58	57	61
Ulno carpal pain and prior trauma					
Ulnar fovea sign	90	8	54	55	39
Ulnar grinding test	93	12	57	57	57
MRI	93	17	59	58	65
Palmar A1 lesion					
Ulnar fovea sign	86	9	40	39	50
Ulnar grinding test	90	12	42	39	66
MRI	90	23	48	41	79
Palmer 2c Lesion					
Ulnar fovea sign	85	9	26	22	68
Ulnar grinding test	93	12	30	23	86
MRI	88	23	37	24	87

For peer review only

PRISMA 2009 Checklist

Section/topic	#	Checklist item	Reported on page #
**TITLE**			
Title	1	Identify the report as a systematic review, meta-analysis, or both.	1
**ABSTRACT**			
Structured summary	2	Provide a structured summary including, as applicable: background; objectives; data sources; study eligibility criteria, participants, and interventions; study appraisal and synthesis methods; results; limitations; conclusions and implications of key findings; systematic review registration number.	2
**INTRODUCTION**			
Rationale	3	Describe the rationale for the review in the context of what is already known.	4
Objectives	4	Provide an explicit statement of questions being addressed with reference to participants, interventions, comparisons, outcomes, and study design (PICOS).	4-5
**METHODS**			
Protocol and registration	5	Indicate if a review protocol exists, if and where it can be accessed (e.g., Web address), and, if available, provide registration information including registration number.	6
Eligibility criteria	6	Specify study characteristics (e.g., PICOS, length of follow-up) and report characteristics (e.g., years considered, language, publication status) used as criteria for eligibility, giving rationale.	7
Information sources	7	Describe all information sources (e.g., databases with dates of coverage, contact with study authors to identify additional studies) in the search and date last searched.	7
Search	8	Present full electronic search strategy for at least one database, including any limits used, such that it could be repeated.	7
Study selection	9	State the process for selecting studies (i.e., screening, eligibility, included in systematic review, and, if applicable, included in the meta-analysis).	7
Data collection process	10	Describe method of data extraction from reports (e.g., piloted forms, independently, in duplicate) and any processes for obtaining and confirming data from investigators.	8
Data items	11	List and define all variables for which data were sought (e.g., PICOS, funding sources) and any assumptions and simplifications made.	
Risk of bias in individual studies	12	Describe methods used for assessing risk of bias of individual studies (including specification of whether this was done at the study or outcome level), and how this information is to be used in any data synthesis.	8
Summary measures	13	State the principal summary measures (e.g., risk ratio, difference in means).	
Synthesis of results	14	Describe the methods of handling data and combining results of studies, if done, including measures of consistency (e.g., I^2) for each meta-analysis.	

For peer review only

For peer review only - http://bmjopen.bmj.com/site/about/guidelines.xhtml

PRISMA 2009 Checklist

Section/topic	#	Checklist item	Reported on page #
Risk of bias across studies	15	Specify any assessment of risk of bias that may affect the cumulative evidence (e.g., publication bias, selective reporting within studies).	
Additional analyses	16	Describe methods of additional analyses (e.g., sensitivity or subgroup analyses, meta-regression), if done, indicating which were pre-specified.	8
**RESULTS**			
Study selection	17	Give numbers of studies screened, assessed for eligibility, and included in the review, with reasons for exclusions at each stage, ideally with a flow diagram.	9
Study characteristics	18	For each study, present characteristics for which data were extracted (e.g., study size, PICOS, follow-up period) and provide the citations.	9
Risk of bias within studies	19	Present data on risk of bias of each study and, if available, any outcome level assessment (see item 12).	12
Results of individual studies	20	For all outcomes considered (benefits or harms), present, for each study: (a) simple summary data for each intervention group (b) effect estimates and confidence intervals, ideally with a forest plot.	14-19
Synthesis of results	21	Present results of each meta-analysis done, including confidence intervals and measures of consistency.	
Risk of bias across studies	22	Present results of any assessment of risk of bias across studies (see Item 15).	
Additional analysis	23	Give results of additional analyses, if done (e.g., sensitivity or subgroup analyses, meta-regression [see Item 16]).	20-21
**DISCUSSION**			
Summary of evidence	24	Summarize the main findings including the strength of evidence for each main outcome; consider their relevance to key groups (e.g., healthcare providers, users, and policy makers).	21-24
Limitations	25	Discuss limitations at study and outcome level (e.g., risk of bias), and at review-level (e.g., incomplete retrieval of identified research, reporting bias).	3
Conclusions	26	Provide a general interpretation of the results in the context of other evidence, and implications for future research.	24
**FUNDING**			
Funding	27	Describe sources of funding for the systematic review and other support (e.g., supply of data); role of funders for the systematic review.	26

From: Moher D, Liberati A, Tetzlaff J, Altman DG, The PRISMA Group (2009). Preferred Reporting Items for Systematic Reviews and Meta-Analyses: The PRISMA Statement. PLoS Med 6(7): e1000097. doi:10.1371/journal.pmed1000097

For more information, visit: **www.prisma-statement.org**.

For peer review only - http://bmjopen.bmj.com/site/about/guidelines.xhtml

BMJ Open

Diagnostic accuracy of history taking, physical examination and imaging for non-chronic finger, hand and wrist ligament and tendon injuries: a systematic review update.

Journal:	*BMJ Open*
Manuscript ID	bmjopen-2020-037810.R1
Article Type:	Original research
Date Submitted by the Author:	06-Jul-2020
Complete List of Authors:	Krastman, Patrick; Erasmus Medical Center,<br>Mathijssen, Nina; Reinier de Graaf Gasthuis, Orthopaedic<br>Bierma-Zeinstra, Sita; Erasmus University Medical Centre, Department of General Practice<br>Kraan, Gerald; Reinier de Graaf Hospital, Orthopeadic<br>Runhaar, Jos; Erasmus University Medical Center Rotterdam, Department of General Practice
**Primary Subject Heading**:	Diagnostics
Secondary Subject Heading:	Radiology and imaging
Keywords:	Diagnostic radiology < RADIOLOGY & IMAGING, Hand & wrist < ORTHOPAEDIC & TRAUMA SURGERY, Adult orthopaedics < ORTHOPAEDIC & TRAUMA SURGERY

SCHOLARONE™
Manuscripts

I, the Submitting Author has the right to grant and does grant on behalf of all authors of the Work (as defined in the below author licence), an exclusive licence and/or a non-exclusive licence for contributions from authors who are: i) UK Crown employees; ii) where BMJ has agreed a CC-BY licence shall apply, and/or iii) in accordance with the terms applicable for US Federal Government officers or employees acting as part of their official duties; on a worldwide, perpetual, irrevocable, royalty-free basis to BMJ Publishing Group Ltd ("BMJ") its licensees and where the relevant Journal is co-owned by BMJ to the co-owners of the Journal, to publish the Work in this journal and any other BMJ products and to exploit all rights, as set out in our licence.

The Submitting Author accepts and understands that any supply made under these terms is made by BMJ to the Submitting Author unless you are acting as an employee on behalf of your employer or a postgraduate student of an affiliated institution which is paying any applicable article publishing charge ("APC") for Open Access articles. Where the Submitting Author wishes to make the Work available on an Open Access basis (and intends to pay the relevant APC), the terms of reuse of such Open Access shall be governed by a Creative Commons licence – details of these licences and which Creative Commons licence will apply to this Work are set out in our licence referred to above.

Other than as permitted in any relevant BMJ Author's Self Archiving Policies, I confirm this Work has not been accepted for publication elsewhere, is not being considered for publication elsewhere and does not duplicate material already published. I confirm all authors consent to publication of this Work and authorise the granting of this licence.

For peer review only

Diagnostic accuracy of history taking, physical examination and imaging for non-chronic finger, hand and wrist ligament and tendon injuries: a systematic review update.

Patrick Krastman MD[a], Nina M. Mathijssen PhD[c], Sita M.A. Bierma-Zeinstra PhD[a,b], Gerald Kraan PhD[c], Jos Runhaar PhD[a]

[a] Erasmus MC University Medical Center, Rotterdam, the Netherlands, Department of General Practice.

[b] Erasmus MC University Medical Center, Rotterdam, the Netherlands, Department of Orthopedics.

[c] Reinier de Graaf Groep, Delft, the Netherlands, Department of Orthopedic Surgery.

Patrick Krastman MD (corresponding author): wetenschap@dezorghoek.nl, +31(0)10-7044192

Nina M. Mathijssen PhD: N.Mathijssen@rdgg.nl, +31(0)15-2604863

Sita M.A. Bierma-Zeinstra PhD: s.bierma-zeinstra@erasmusmc.nl, +31(0)10-7044192

Gerald Kraan PhD: KraanG@rdgg.nl, +31(0)15-2604863

Jos Runhaar PhD: j.runhaar@erasmusmc.nl, +31(0)10-7044192

Keywords: wrist, hand, finger, diagnostic tests, traumatic injury, ligament.

For peer review only - http://bmjopen.bmj.com/site/about/guidelines.xhtml

Abstract

Objective: The diagnostic work-up for ligament and tendon injuries of the finger, hand and wrist consists of history taking, physical examination and imaging if needed, but the supporting evidence is limited. The main purpose of this study was to systematically update the literature for studies on the diagnostic accuracy of tests for detecting non-chronic ligament and tendon injuries of the finger, hand and wrist.

Methods: Medline, Embase, Cochrane Library, Web of Science, Google scholar ProQuest and Cinahl were searched from 2000 up to 6 February 2019 for identifying studies. Methodological quality was assessed using the QUADAS-2 checklist and sensitivity (Se), specificity (Sp), accuracy, positive predictive value (PPV) and negative predictive value (NPV) were extracted.

Results: None of the studies involved history taking. Physical examination, for diagnosing lesions of the triangular fibrocartilage complex (TFCC), showed Se, SP, accuracy, PPV and NPV ranging from 58-90%, 20-69%, 56-73%, 53-71% and 55-65%, respectively. Physical examination in hand and finger injuries the Se, Sp, accuracy, PPV and NPV ranged from 88 to 99%, 75 to 100%, 34 to 88%, 91 to 100% and 75 to 95%, respectively. The accuracy of Magnetic Resonance Imaging (MRI) with high-resolution (3 tesla) techniques for TFCC and interosseous ligaments of the proximal carpal row ranged from 89-91% and 75-100%, respectively. The accuracy of MRI with low-resolution (1.5 tesla) techniques for TFCC and interosseous ligaments of the proximal carpal row ranged from 81-100% and 67-95%, respectively.

Conclusions:

There is limited evidence on the diagnostic accuracy of history taking and physical examination for non-chronic finger, hand and wrist ligament and tendon injuries. Although some imaging modalities seemed to be acceptable for the diagnosis of ligament and tendon

For peer review only - http://bmjopen.bmj.com/site/about/guidelines.xhtml

injuries in the wrist in patients presenting to secondary care, there is no evidence-based advise possible for the diagnosis of non-chronic finger, hand, or wrist ligament and tendon injuries in primary care.

Strengths and Limitations of this study:

- This is the first study that systematically reviewed the accuracy of diagnostic tests for non-chronic hand and finger injuries, next to previously described accuracy of diagnostic tests for non-chronic wrist injuries.
- Studies on wrist injuries published before 2000 were not evaluated and not included in the current systematic review, as these were adequately described in published systematic reviews.
- Diagnostic tests heterogeneity precluded meta-analysis, caused by the fact that studies that evaluated the same pathologies showed marked diversity in population, index tests, reference test and methodological quality.

For peer review only - http://bmjopen.bmj.com/site/about/guidelines.xhtml

Introduction

Wrist injuries are one of the most common presentations to the emergency department due to trauma and they commonly affect young people of working age.[1 2] In the Netherlands, 21 per cent of the patients initially consulted their general practitioner (GP) after a wrist injury, 41 per cent went directly to an outpatient clinic and 35 per cent had no further treatment.[3] Within the GP's practice, the prevalence of hand injuries is 10 for each 1000 patients per year, while the prevalence for wrist injuries is six for each 1000 patients per year.[4] In an Emergency Department (ED) injuries to the hand and wrist are common and they account for between 10% and 30% of all presentations.[3 5 6 7] Traumatic hand injuries are a frequent part among work-related injuries and can result in prolonged sick-leave. They represent a considerable economic burden, with both high health-care and productivity costs.[5] If not treated properly, patients may experience lifelong pain and functional limitations that have major effects on the quality of life and could result in patients losing their jobs.[8]

The standard diagnostic work-up for non-chronic finger, hand and wrist trauma consists of history taking, a physical examination and, if needed, imaging. There is general agreement that a detailed patient history and a conscientious clinical examination should be standard methods of diagnosing wrist pain.[9] Nevertheless, the diagnosis of wrist pathologies remains complex and challenging, since the wrist contains many joints that function together to move the hand, and there is increasing demand for evidence for diagnostic technologies, such as imaging tools.[10]

Evidence-based medicine is required to create well-founded policies for non-chronic finger, hand and wrist ligament and tendon injuries. It is essential to distinguish between diagnosing these injuries in hospital care and in non-institutionalized GP care, as results from diagnostic studies in hospital care cannot automatically be translated into guidelines for non-institutionalized GP care.[11] Diagnostic accuracy is affected by the prevalence of the

For peer review only - http://bmjopen.bmj.com/site/about/guidelines.xhtml

pathology. Predictive values are largely dependent on the prevalence of the pathology in the examined population. Therefore, predictive values from one study should not be transferred to another setting with a different prevalence of the disease in the population.[12] Nevertheless, currently available systematic reviews on the diagnostic accuracy of tests for the diagnosis of finger, hand and wrist pathologies did not distinguish between hospital and non-institutionalized GP care settings when presenting their results.[10 13-15] Within the available systematic reviews, published up to 2015, no studies were found on the diagnostic accuracy of history taking and only the scaphoid shift test and high-resolution MRI were recommended for diagnosing TFCC tears.[10 13-15]

The main purpose of the present study was to provide a systematic overview of the diagnostic accuracy of history taking, physical examination and imaging for detecting non-chronic ligament and tendon injuries of the finger, hand and wrist. The secondary aim of this study was to retrieve the clinical care setting (hospital or non-institutionalized GP) of the eligible studies and the studies published in previous systematic reviews.

Methods

The Preferred Reporting Items for Systematic Reviews and Meta-Analyses (PRISMA) Statement was used to guide the conduct and reporting of the study.[16] A review protocol was composed prior to searching the literature, but central registration was not completed.

Search strategy

A Biomedical Information specialist (Wichor M. Bramer) from the Medical Library at Erasmus MC performed a search for studies in Medline, Embase, Cochrane Library, Web of Science, Google scholar ProQuest and Cinahl from 2000 up to 6 February 2019. This starting point was used since multiple reviews are available that already cover the period up to the year 2000 (Table 1). Search terms included hand, finger and wrist injuries, history taking,

For peer review only - http://bmjopen.bmj.com/site/about/guidelines.xhtml

provocative test(s), diagnostic test(s) and imaging tests. The full electronic search strategy for the Embase database is presented in Appendix 1.

Study Selection criteria

Studies describing diagnostic accuracy of history taking, physical examination or imaging in adult patients (age ≥ 16 years) with non-chronic finger, hand and wrist ligament and tendon injuries were included. Diagnostic accuracy was reported or could be calculated. Case reports, reviews and conference proceedings were excluded. Distal radius and ulna injuries were also excluded. Chronic injuries (e.g. osteoarthritis) were excluded as a result of another pathophysiology.There was no gold-standard reference test against which to assess history taking, physical examination or imaging measurements. Surgical observations (arthroscopy) are the reference standard for confirming a diagnosis of non-chronic hand, finger or wrist injury, although only a subset of patients suspected of having non-chronic hand, finger or wrist injury require surgery. To decrease verification bias, diagnostic-imaging techniques for non-chronic hand injury were accepted as reference tests as well. Since tendinopathy does not typically require surgery, imaging is also a pragmatic reference standard for this condition. As this review focused on non-chronic pathologies, studies including patient with chronic pathologies (e.g. osteoarthritis, rheumatic arthritis) were excluded. Infection and neurologic injuries are out of the scope of this review and are therefore not included. Carpal tunnel syndrome is extensively described in the literature and was therefore not included in this review.[17-19] Diagnoses of musculoskeletal soft-tissue tumours were also excluded. No language restrictions were applied. For languages of the eligible studies other than English, Google translate was used for the first translation of these studies. If necessary, a professional translator was consulted.[20]

Two reviewers (PK, YA) read all titles and abstracts independently. Articles that could not be excluded on the basis of the title and/or abstract were retrieved in full text and were read and

For peer review only - http://bmjopen.bmj.com/site/about/guidelines.xhtml

checked for inclusion by the two reviewers independently. If there was no agreement, a third reviewer (JR) made the final decision. In addition, the reference lists of all included studies were reviewed to check for additional relevant studies.

Data collection process and Methodological Quality Assessment

In the current review our primary outcome measures were the positive predictive value (PPV) and the negative predictive value (NPV) of diagnostic tests. Secondary outcome measure were the sensitivity, specificity and accuracy of diagnostic tests.

Two reviewers (PK, JR) independently extracted the data. Data were extracted describing the study design, characteristics of the study population, test characteristics, setting (hospital care or non-institutionalized GP care), and diagnostic parameters. The following values were extracted, when documented: sensitivity (Se), specificity (Sp), accuracy, positive predictive value (PPV) and negative predictive value (NPV). If diagnostic parameters were not reported, they were calculated from reported data or authors were contacted by email when data was unavailable. The following formula was used, when calculating diagnostic accuracy: Diagnostic accuracy = No. of true positives + no. of true negatives / Total no. of subjects.[21] If an included study presented results from multiple independent observers, accuracy measures were averaged over the observers. Furthermore, data of the studies published in previous systematic reviews were extracted describing the setting (hospital care or non-institutionalized GP care). Methodological quality was assessed using the Quality Assessment of Diagnostic Accuracy Studies (QUADAS-2) checklist.[22] This tool allows more transparent rating of bias and applicability in primary diagnostic accuracy studies. The QUADAS-2 tool consists of four domains: patient selection, index test, reference standard, and flow and timing. Two reviewers (PK, JR) independently assessed the risk of bias and applicability of each included study. Disagreements were resolved by discussion. Questions were answered with ‘yes’, ‘no’ or ‘unclear’.

For peer review only - http://bmjopen.bmj.com/site/about/guidelines.xhtml

Patient and public involvement

Patients and members of the public were not involved in this systematic review update.

Results

Study Selection

The flow diagram for the categorization process is presented in Figure 1. We assessed 209 full-text articles for eligibility out of 4867 records identified through database searches. A total of 23 diagnostic studies were finally identified, assessed and interpreted.

Study characteristics

The characteristics of the studies are presented in Table 1.

For peer review only

Table 1. Characteristics of the Eligible Studies (N=23)

Author (year)	Participants	Design	Setting (Country)	Trauma	Index test 1	Index test 2	Reference test
**Wrist injuries**							
Anderson[23] (2008)	102	Retrospective	Not described (USA)	TFCC/SLIL /LTIL/UTIL	MRI 1,5T	MRI 3T	Arthroscopy
Pahwa[24] (2014)	53	Prospective	Not described (India)	TFCC/SLIL/LTIL	MRI 1,5T	MR arthrography	Arthroscopy
Prosser[25] (2011)	105	Prospective	Private hand clinic (Australia)	TFCC/SLIL/LTIL	MRI 1T	Provocative tests	Arthroscopy
Langner[26] (2015)	38	Not described	Not described (Germany)	SL dissociation	Cine MRI 3T	Cineradiography	Arthroscopy
Spaans[27] (2013)	37	Not described	Department for Hand and Plastic Surgery # (Netherlands)	SLIL (complete tear)	MRI 3T		Arthrotomy
Greditzer[28] (2016)	26	Retrospective	Department for Hand and Plastic Surgery# (USA)	SLIL	MRI 1,5T axial sequences	MRI 1,5T coronal sequences	Arthroscopy
Al-Hiari[29] (2013)	42	Prospective	Orthopedic Surgery# (Jordan)	TFCC (full-thickness tears)	MR arthrography		Arthroscopy
Schmauss[30] (2016)	908	Retrospective	Department for Hand and Plastic Surgery (Germany)	TFCC	MRI (resolution not described)	Provocative tests	Arthroscopy
Lee[31] (2016)	39	Prospective	Not described (China)	TFCC (full-thickness tears)/SLIL/LTIL	MR (3T) arthrography without traction	MR (3T) arthrography with traction	Conventional arthrography
Finlay[32] (2004)	26	Retrospective	Not described (Canada)	TFCC/SLIL/LTIL	US (9-13MHz)		MR arthrography**
Dornberger[33] (2015)	72	Prospective	Hand surgery# (Germany)	SLIL	Radiographs		Arthroscopy
Koskinen[34] (2012)	52	Not described	Not described (Finland)	TFCC/ SLIL/ LTIL	CBCT arthrography*		MR arthrography
Boer[35] (2018)	150	Retrospective	Plastic or Orthopedic Surgery (Netherlands)	TFCC	MRI 1,5 or 3,0T	MRA 1,5 or 3,0T	Arthroscopy
Lee[36] (2018)	65	Prospective	Emergency department (Korea)	TFCC	US		MRI 3,0T
Suojärvi[37] (2017)	21	Prospective	Hand surgery (Finland)	SLIL/LTIL/TFCC	CBCT arthrography	MRA	Arthroscopy
Mahmood[38] (2012)	30	Retrospective	General hospital (UK)	SLIL/LTIL/TFCC	MRA		Arthroscopy
**Hand and finger injuries**							

For peer review only

Lutsky[39] (2014)	20	Retrospective	Not described (USA)	Collateral ligament tears of the MPJ of the fingers	MRI (open,1,5T, 3T)		Surgical findings
Guntern[40] (2007)	8	Retrospective	Not described (Switzerland)	A2 pulley Lesion	Clinical examination		MRI 3T
Klauser[41] (2002)	64	Not described	Not described (Austria)	Finger pulley injuries	US (12MHz)		MRI 1,5T
Lee[42] (2000)	10	Not described	Not described (USA)	Flexor tendon injuries	US (L10-5MHz)		Surgical findings
Zhang[43] (2012)	92	Not described	Department of Surgery (China)	Flexor tendon injuries	US (10MHz)		Surgical findings
Mahajan[44] (2016)	30	Prospective	Emergency Room and outpatients clinic of Surgery and Orthopedics (Netherlands)	UCL injuries	Clinical examination		MRI 1,5T
Shekarchi[45] (2017)	20	Prospective	Emergency department (Iran)	UCL of the thumb	US		MRI

* Cone Beam CT arthrography

** Tricompartment wrist arthrography

setting for the study was obtained after email contact

TFCC: Triangular Fibrocartilage Complex
SLIL: Scapholunate Interosseous Ligament
UTIL: Ulnotriquetral Interosseous Ligament
LTIL: Lunotriquetral Ligament
UCL: Ulnar Collateral Ligament

For peer review only

Eight studies were retrospective[23 28 30 32 35 36 39 40], nine studies were prospective[24 25 29 31 33 36 37 45 46] and six studies[26 27 34 37-39] gave no description of the study design. Eight studies[23 25 30 33 35 36 41 42] included more than 60 participants; six of these studies[23 25 30 33 35 36] described wrist pathologies and two[41 42] described hand pathologies. In total, 16 studies[23-38] described injuries to the wrist anatomy and seven studies[39-45] described injuries to the hand/finger anatomy.

Quality Assessment

There was considerable underreporting of important quality domains in most studies (see Table 2).

Table 2. Summary of Methodological Quality According to Quality Assessment of Diagnostic Accuracy Studies-2.

**Author (year), index test(s)**	**Risk of Bias**				**Applicability Concerns**		
	Patient Selection	Index Test	Reference standard	Flow and Timing	Patient Selection	Index Test	Reference standard
**Wrist disabilities**							
Anderson[23] (2008)	LR	LR	HR	LR	LR	LR	LR
Pahwa[24] (2014)	UR	LR	HR	HR	LR	LR	LR
Prosser[25] (2011) Provocative tests	LR	LR	LR	LR	LR	LR	LR
MRI	LR	LR	HR	LR	LR	LR	LR
Langner[26] (2015)	LR	LR	HR	HR	LR	LR	LR
Spaans[27] (2013)	UR	LR	LR	UR	LR	LR	LR
Greditzer[28] (2016)	HR	LR	HR	LR	LR	LR	LR
Al-Hiari[29] (2013)	LR	LR	HR	LR	LR	LR	LR
Schmauss[30] (2016)	LR	HR	HR	LR	LR	LR	LR
Lee[31] (2016)	LR	HR	HR	LR	LR	LR	LR
Finlay[32] (2004)	UR	LR	LR	LR	LR	LR	LR
Dornberger[33] (2015)	LR	LR	HR	LR	LR	LR	LR
Koskinen[34] (2012)	LR	HR	HR	LR	LR	LR	LR

For peer review only

Boer[35] (2018)	HR	UR	HR	LR	LR	LR	LR
Lee[36] (2018)	LR	LR	LR	LR	LR	LR	LR
Suojärvi[37] (2017)	LR	LR	HR	HR	LR	LR	LR
Mahmood[38] (2012)	UR	LR	UR	LR	LR	LR	LR
**Hand and finger disabilities**							
Lutsky[39] (2014)	LR	UR	HR	LR	LR	LR	LR
Guntern[40] (2007)	LR	HR	LR	LR	LR	LR	LR
Klauser[41] (2002)	LR	HR	LR	HR	LR	LR	LR
Lee[42] (2000)	UR	LR	LR	LR	LR	LR	LR
Zhang[43] (2012)	LR	LR	HR	LR	LR	LR	LR
Mahajan[44] (2016)	LR	LR	LR	LR	LR	LR	LR
Shekarchi[45] (2017)	UR	LR	LR	LR	LR	LR	LR
Abbreviations:	LR: Low Risk	HR: High Risk	U: Unclear Risk				

Two studies had low risk of bias on all quality domains.[25 36] In eight[24 27 28 32 35 38 42 45] of the 23 studies, patient selection was not well documented. Furthermore, the risk of bias was predominantly influenced by the lack of a proper description of the index test (30%, 7/23)[30 31 34 35 39-41] or the reference standard (65%, 15/23)[23-26 28-31 33-35 37-39 43]. Regarding flow and timing, not all patients received the reference standard in four studies (22%, 5/23).[24 27 29 37 41] Due to our selection procedure, all the studies match the review question.

Accuracy of diagnostic tests concerning Wrist injuries

None of the studies evaluated the diagnostic accuracy of history taking. Physical examination was evaluated in 2 studies for diagnosing lesions of the triangular fibrocartilage complex (TFCC).[25 30] Provocative wrist tests for diagnosing scapholunate interosseous ligament (SLIL) and lunotriquetral interosseous ligament (LTIL) lesions was assessed in one study.[25] Radiographs were used as an index test in 1 study for diagnosing SLIL lesions.[33] US for diagnosing TFCC lesions was used in 2 studies.[32 36] Two studies used Cone Beam Computed Tomography (CBCT) as index for diagnosing TFCC lesions.[34 37] In 12 studies MRI was used

For peer review only

as an index test.[23-31 35 37 38] The accuracy of MRI for TFCC, SLIL, LTIL and UTIL lesions with high-resolution (3 tesla) techniques ranging from 89-91%, 75-92%, 91% and 100%, respectively. The accuracy of MRI for TFCC, SLIL, LTIL and UTIL lesions with low-resolution (1.5 tesla) techniques ranging from 81-100%, 67-81%, 81-94% and 95%, respectively. The accuracy measures of the diagnostic tests are presented in table 3.

For peer review only

For peer review only - http://bmjopen.bmj.com/site/about/guidelines.xhtml

Table 3. Accuracy of the Diagnostic Tests of the Wrist

**Author (year)**	**Index test 1**	**Reference test**	**Trauma**	**Se % (95% CI)**	**Sp % (95% CI)**	**Accuracy % (95% CI)**	**PPV % (95% CI)**	**NPV % (95% CI)**
**PHYSICAL EXAMINATION**								
Prosser[25] (2011)	Provocative tests	Arthroscopy	TFCC	58	69	73	71	55
			SLIL	61	79	78	68	74
			LTIL	17	84	95	6	94
Schmauss[30] (2016)	Fovea sign	Arthroscopy	TFCC	73	44	58	53	66
	Ulna grinding test			90	20	56	54	65
**IMAGING: RADIOGRAPHS**								
Dornberger[33] (2015)	Radiographs (Stecher's projection)	Arthroscopy	SLIL	(76.9+80.8)/2**	(86.4+84.1)/2**	(92.7+90.6)/2**	(76.9+75)/2**	(86.4+88.1)/2**
**IMAGING: US**								
Finlay[32] (2004)	US (9-13MHz)	MR arthrography Tricompartment	SLIL	100	100	100	100	100
			TFCC	64	100	85	100	79
			LTIL	25	100	77	100	75
Lee[36] (2018)	US	MRI	TFCC, total	99**	88**	97**	97**	95**
**IMAGING: MRI**								
Anderson[23] (2008)	MRI 1,5T	Arthroscopy	TFCC	82	59	83 (72.4-89.9)*		
			SLIL	57	83	78 (67.2-86.3)*		
			UTIL	57	89	95 (86.1-98.3)*		

For peer review only - http://bmjopen.bmj.com/site/about/guidelines.xhtml

			LTIL	22	94	86 (75.3-91.9)*		
	MRI 3T		TFCC	90	74	91 (75.8-96.8)*		
			SLIL	70	94	91 (75.8-96.8)*		
			UTIL	67	87	100 (97.9-100)*		
			LTIL	50	94	91 (75.8-96.8)*		
Pahwa[24] (2014)	MR arthrography	Arthroscopy	TFCC	100	100	100	100	100
			SLIL	100	100	100	100	100
			LTIL	100	100	100	100	100
	MRI 1,5T MEDIC		TFCC	83	100	81	91	60
			SLIL	63	100	81	100	73
			LTIL	40	100	81	100	73
	MRI FS PD/T2		TFCC	75	100	75	90	50
			SLIL	38	100	69	100	62
			LTIL	20	100	75	100	73
Prosser[25] (2011)	MRI 1T	Arthroscopy	TFCC			86 (PT+MRI)		
			SLIL			80 (PT+MRI)		
			LTIL			94 (PT+MRI)		
Schmauss[30] (2016)	MRI resolution not described	Arthroscopy		76	41	58	55	65
Langner[26] (2015)	Cine MRI 3,0T and Cineradiography	Arthroscopy	SL dissociation	85	90	92		
Spaans[27] (2013)	MRI 3T	Arthrotomy	SLIL	75.5**	100*	75**	98.5**	8*
Greditzer[28] (2016)	MRI 1,5T axial sequences	Arthroscopy	SLIL	79	82	80	76	84

For peer review only - http://bmjopen.bmj.com/site/about/guidelines.xhtml

	MRI 1,5T coronal sequences		SLIL	65	69	67	68	71
Al-Hiari[29] (2013)	MR arthrography	Arthroscopy	TFCC	93	80	85		
Lee[31] (2016)	MR arthrography without traction	Conventional arthrography	TFCC	83	81	83	87	76
			SLIL	66	97	95	67	97
			LTIL	57	94	88	67	91
	MR arthrography with traction		TFCC	96	100	98	100	94
			SLIL	100	100	100	100	100
			LTIL	100	100	100	100	100
Boer[35] (2018)	MRI 1,5T	Arthroscopy	TFCC	71	75	100	71	75
	MRI 3,0T	Arthroscopy	TFCC	73	67	89	83	52
	MR arthrography 1,5T	Arthroscopy	TFCC	80	100	80	100	50
	MR arthrography 3,0T	Arthroscopy	TFCC	73	100	73	100	60
Suojärvi[37] (2017)	MR arthrography	Arthroscopy	SLIL	25 (3-65)	80 (61-92)	68 (51-83)	25 (3-65)	80 (61-92)
			LTIL	50 (7-93)	77 (59-90)	74 (57-88)	22 (3-60)	92 (75-99)
			TFCC	44 (22-69)	50 (25-75)	47 (30-65)	50 (25-75)	44 (21-69)
			SLIL or LTIL	33 (7-60)	79 (67-88)	72 (56-82)	24 (7-50)	86 (74-94)
Mahmood[38] (2012)	MR arthrography	Arthroscopy	SLIL	91	88		83	88
			LTIL	100	100		100	100
			TFCC	90	75		85	80
**IMAGING: COMPUTED TOMOGRAPHY**								
Koskinen[34] (2012)	CBCT arthrography	MR arthrography	TFCC	76	90	87	83	87

For peer review only - http://bmjopen.bmj.com/site/about/guidelines.xhtml

			SLIL	56	91	83	67	89
			LTIL	83	81	82	44	96
Suojärvi[37] (2017)	CBCT	Arthroscopy	SLIL	63 (24-91)	87 (69-96)	82 (66-92)	56 921-86)	90 (73-98)
			LTIL	100 (40-100)	59 (41-76)	64 (46-79)	24 (7-50)	100 (83-100)
			TFCC	67 (40-87)	89 (63-98)	77 (60-90)	86 (57-98)	73 (50-89)
			SLIL or LTIL	75 (43-95)	76 (65-86)	73 (61-83)	35 (16-53)	95 (86-99)

TFCC: Triangular fibrocartilage complex
SLIL: Scapholunate interosseous ligament
UTIL: Ulnotriquetral interosseous ligament
LTIL: Lunotriquetral ligament
CBCT: Cone Beam Computed Tomography
MRI: Magnetic resonance imaging
T: Tesla
US: Ultra Sonography
Se: Sensitivity
Sp: Specificity
PPV: Positive predictive value
NPV: Negative predictive value
n/a: not available due to low prevalence
* Only reported for one of two readers.
** Average between presented individual values of two readers.
*** SL distance 3.7mm on Stecher's projection

For peer review only

In addition to the data presented in Table 3, the study of Schmauss et al. presented the diagnostic accuracy of their tests separately for different subgroups.[30] These results are summarized in Appendix 2.

Accuracy of diagnostic tests concerning Hand and Finger injuries

Table 4 describes the accuracy of the diagnostic test for non-chronic hand and fingers injuries.[39-45]

Table 4. Accuracy of the Diagnostic Tests of the Hand and Fingers.

**Author (year)**	**Index test 1**	**Reference test**	**Trauma**	**Se (%) (95% CI)**	**Sp (%) (95% CI)**	**Accuracy (%) (95% CI)**	**PPV (%) (95% CI)**	**NPV (%) (95% CI)**
Lutsky[39] (2014)	MRI (open,1,5Tor 3T)	Surgical findings	Collateral ligament tears of the MPJ of the fingers	64	∞	64	100	∞
Guntern[40] (2007)	Clinical examination	MRI 3T	A2 pulley Lesion	88	100	88	100	95
Klauser[41] (2002)	US (12MHz)	MRI 1,5T (and Surgical findings, n=7)	Finger pulley injuries	98	100	99	100	97
Lee[42] (2000)	US (10-5MHz)	Surgical findings	Flexor tendon injuries			90		
Zhang[43] (2012)	US (10MHz)	Surgical findings	Flexor tendon injuries			100		
	History and Clinical examination					34		
Mahajan[44] (2016)	Clinical examination	MRI 1,5T	UCL injuries	91	75	87	91	75
Shekarchi[45] (2017)	US	MRI	UCL of the thumb	71 (30-95)	85 (54-97)	80	71 (30-95)	85 (54-97)

MRI: Magnetic resonance imaging

T: Tesla

US: Ultra Sonography

UCL: Ulnar Collateral Ligament

For peer review only

Se:	Sensitivity
Sp:	Specificity
PPV:	Positive predictive value
NPV:	Negative predictive value
∞:	Infinity

Two studies concerned flexor tendon injuries[42 43], while the other studies concerned collateral ligament tears of the metacarpophalangeal Joint (MPJ) of the fingers[39], A2 pulley lesions[40], finger pulley injuries[41] and ulnar collateral ligament injuries[44 45]. None of the studies involved history taking. Clinical examination was used three times as an index test.[40 43 44] The Se, Sp, accuracy, PPV and NPV of physical examination in hand and finger injuries ranged from 88 to 99%, 75 to 100%, 34 to 88%, 91 to 100% and 75 to 95%, respectively. MRI was used once as an index test.[39] Four studies used ultrasonography (US) as an index test.[41-43 45] The accuracy of US in flexor injuries ranged from 90 to 100%.[42 43] The accuracy of US for finger pulley injuries and Ulnar Collateral Ligament (UCL) of the thumb was 99% and 80%, respectively.[41 45]

Clinical care setting

The clinical care setting was described in 9 out of 23 studies and was obtained by contacting the authors for an additional 4 studies: a private hand clinic[25], emergency department[36 45], department for hand and plastic surgery[27 28 29 33 35], surgery[43], orthopaedics department[29 35] and in an emergency room and outpatient clinic of a surgery and orthopaedics department[44]. Despite multiple attempts to contact the authors by email, clarification regarding the setting could not be obtained for the remaining 10 studies.

For peer review only

For peer review only - http://bmjopen.bmj.com/site/about/guidelines.xhtml

Discussion

The standard diagnostic work-up for non-chronic finger, hand and wrist trauma consists of history taking, a physical examination and, if needed, imaging. There is general agreement that a detailed patient history and a conscientious clinical examination should be standard methods of diagnosing wrist pain.[9] Our systematic review showed that there still is a gap in knowledge regarding valid diagnostic tests for non-chronic wrist ligament and tendon injuries. Moreover, for the first time, the lack of high-quality evidence for the diagnosis of ligament and tendon injuries in the hand and fingers has been highlighted in the current systematic overview of the literature.

Previous reviews showed that high-resolution MRI was an accurate means for diagnosing TFCC tears and MRI was slightly specific for tears of the intrinsic ligament, but its sensitivity is low. [10 14] Current review showed that the accuracy measures for MRI showed a wide range in diagnostic outcome values, with diagnostic accuracy measures no better for high-resolution MRI. The present results indicate that the accuracy for tears of the TFCC, SLIL and LTIL is increased by MRA.

Diagnostic Accuracy of the Diagnostic Tests of the Wrist

Although common practice in hospital care, in previous reviews[10 13-15] and current systematic review update, no studies were identified on the diagnostic accuracy of history taking for non-chronic ligament and tendon injuries of the wrist.

This systematic review update included one new study on physical examinations for diagnosing non-chronic ligament and tendon injuries of the wrist, which did not affect the previous conclusion that physical examination is of limited value for diagnosing non-chronic ligament and tendon injuries of the wrist.[30]

For peer review only - http://bmjopen.bmj.com/site/about/guidelines.xhtml

In previous reviews, only the diagnostic performance for MRI and/or MR Arthrography (MRA) of the wrist were examined. This showed that the accuracy of MRI diagnoses of tears of the TFCC was fairly satisfactory (PPV ranged from 71% to 100% and NPV ranged from 37% to 90% for TFCC, PPV ranged from 25% to 100% and NPV ranged from 72% to 94% for SL ligament, and PPV ranged from 0% to 100% and NPV ranged from 74% to 95% for LT ligament) and the best with high-resolution techniques. Contrary, sensitivity, specificity, and accuracy were low for diagnosing intrinsic carpal ligaments injuries (SL and LT), using high-resolution techniques.[10 14] MRA, rather than MRI, was recommended to be used in daily practise for the diagnosis of TFCC injuries.[10 15] In current review, the accuracy measures for MRI showed a wide range in diagnostic outcome values, with diagnostic accuracy measures no better for imaging at 3 tesla than at 1.5 tesla. As previously shown for full-thickness TFCC injuries, the present results indicate that the accuracy for tears of the TFCC, SLIL and LTIL is increased by MRA.[15] Computed tomography (CT) arthrography is an alternative in patients when MRI is contraindicated or when MRI is not available.[34]

In current review, 5 studies used another imaging tool, namely radiograph[33], US[32 36] and CBCT[34 37] for diagnosing non-chronic ligament and tendon injuries of the wrist. The diagnostic accuracy of radiograph was limited. Examination of SLIL and TFCC with US showed promising results and the added value should be further explored. Based on the included studies, CBCT has no added value in assessing non-chronic ligament and tendon injuries of the wrist, especially when we take the methodological quality of the studies into account.

However, a dynamic 4D-CT for the detection of SLIL or LTIL injuries is promising.[47 48] Nevertheless, the diagnostic accuracy has not yet been studied. At present, there is still insufficient scientific evidence regarding the ideal imaging technique for non-chronic intrinsic carpal ligament injuries of the wrist.

For peer review only - http://bmjopen.bmj.com/site/about/guidelines.xhtml

In current systematic review update and previous systematic reviews, the reported diagnostic accuracy measures for imaging modalities were characterized by markedly heterogeneous results. It was not appropriate to pool results for the diagnostic accuracy of imaging, due to a lack of multiple imaging studies on one specific wrist injury. Based on previews and current review we can conclude that MRA rather than MRI is the preferred imaging tool in hospital care setting for detecting non-chronic ligament and tendon injuries of the wrist. The current review focused on diagnostic tests and not on the treatment options for wrist complaints. Arthroscopy, as diagnostic tool, was one of the reference standards in this systematic review. In our opinion is it essential that readily accessible and relatively inexpensive, non-invasive diagnostics are available to and are preferred by clinicians. For some wrist complaints, arthroscopy may be the preferred diagnostic option. However, it is more expensive and invasive than MRI. For that reason diagnostic arthroscopy should be applied with caution, unless a patient is suspected of having non-chronic hand, finger or wrist injury and require therapeutic intervention. The advantage of arthroscopy above MRI is the dynamic modality.

Diagnostic Accuracy of the Diagnostic Tests of the Hand and the Fingers

According to our knowledge, there are no reviews previously published to date on the diagnostic accuracy of history taking, physical examination and imaging for non-chronic ligament and tendon injuries of the finger and hand.

We identified 3 studies on the diagnostic accuracy of history taking and/or clinical examination.[40 43 44] One study[44] had no methodological limitation, while the other two studies had methodological flaws (high risk of bias) on index test[40] and reference standard[43]. In addition, each study evaluated different diagnostics tests for different pathologies. So there is limited evidence on the diagnostic accuracy of history taking and physical examination for diagnosing hand and finger injuries.

For peer review only - http://bmjopen.bmj.com/site/about/guidelines.xhtml

Imaging studies examined a wide variety of imaging tools and pathologies. Moreover, studies with imaging tools as a diagnostic modality had methodological flaws and serious limitations, so we have to interpret these results with caution. Only the study of Lee et al. had relatively few methodological flaws.[42] These authors showed that ultrasonography can possibly help to evaluate completely lacerated flexor tendon injuries. Nevertheless, as indicated by the authors, ultrasonography cannot accurately determine the status of partially transected tendons.[42] The reported diagnostic accuracy measures for imaging modalities were characterized by markedly heterogeneous results. It was not appropriate to pool results for the diagnostic accuracy of imaging, due to the limited number of studies on one specific hand or finger injury and because of the diversity among the eligible studies.

Clinical care setting

The secondary aim of this study was to include the clinical care setting (hospital or non-institutionalized GP) of the eligible studies and the studies published in previous systematic reviews. We assume that all studies included in the current and previous reviews were done in a hospital care setting; this was either described in the paper, was confirmed by the authors, or due to the fact that all authors of the remaining studies were only affiliated to hospitals.

It is essential to distinguish between diagnosing these injuries in hospital care and in non-institutionalized GP care, as results from diagnostic studies in hospital care cannot automatically be translated into guidelines for non-institutionalized GP care.[11] Since previous systematic reviews and the current update of the literature did not identify any studies performed in non-institutionalized GP care, it is not possible to advise GPs with certainty based on the available evidence. Given the burden of non-chronic hand and wrist trauma in non-institutionalized GP care, diagnostic studies focusing on non-chronic hand, finger and wrist ligament and tendon injuries are urgently needed.[1 2]

For peer review only - http://bmjopen.bmj.com/site/about/guidelines.xhtml

Conclusions:

Our systematic review showed that there still is a gap in knowledge regarding valid diagnostic tests for non-chronic wrist ligament and tendon injuries. For the first time, the lack of high-quality evidence for the diagnosis of ligament and tendon injuries in the hand and fingers has been highlighted. Although some imaging modalities seemed to be acceptable for the diagnosis of ligament and tendon injuries in the wrist in patients presenting to secondary care, there are limited tools for adequate diagnosis available to general practitioners. If not diagnosed and treated properly, patients may experience lifelong pain and functional limitations that have major effects on the quality of life and could result in patients losing their jobs.

Abbreviations

CBCT: Cone Beam Computer Tomography

GRIT: Griping rotatory impaction test

HR: High Risk (QUADAS-2)

LR: Low Risk (QUADAS-2)

LTIL: Lunotriquetral interosseous ligament

MRA: Magnetic Resonance Arthrography

MRI: Magnetic Resonance Imaging

NPV: Negative predictive value

PPV: Positive predictive value

Se: Sensitivity

Sp: Specificity

SLIL: Scapholunate interosseous ligament

T: Tesla

For peer review only - http://bmjopen.bmj.com/site/about/guidelines.xhtml

TFCC: Triangular fibrocartilage complex

UCL: Ulnar Collateral Ligament

UR: Unclear Risk (QUADAS-2)

US: Ultra Sonography

UTIL: Ulnotriquetral Interosseous Ligament

Acknowledgement

The authors thank Wichor Bramer (Biomedical information specialist of Erasmus University Medical Center Rotterdam Medical Library) for help with the electronic search strategies and Yassine Aaboubout (MSc) for helping with study selection and extracting the data.

Authors' contributions

PK, NM, SB, GK and JR all contributed to the design of the study. PK and JR were responsible for article selection and analysed the data. All authors contributed to writing and revision of the manuscript. All authors have given approval of the submitted version of the manuscript and agree to be accountable for all aspects of the work.

Funding

No funding.

Availability of data and materials

The datasets used and/or analysed during the current study are available from the corresponding author on reasonable request.

For peer review only - http://bmjopen.bmj.com/site/about/guidelines.xhtml

Ethics approval and consent to participate

Not applicable.

Consent for publication

Not applicable.

Competing interests

The authors declare that they have no competing interests.

For peer review only

REFERENCES

1. Owen RA, Melton III LJ, Johnson KA, Ilstrup DM, Riggs BL. Incidence of Colles' fracture in a North American community. Am J Public Health 1982;72:605–7.
2. McCullough NP, Smith FW, Cooper JG. Early MRI in the management of the clinical scaphoid fracture. Eur J Emerg Med. 2011;18(3):133–6.
3. Larsen CF, Mulder S, Johansen AM, Stam C. The epidemiology of hand injuries in The Netherlands and Denmark. Eur J Epidemiol. 2004;19(4):323-7.
4. MW van der Linden, GP Westert, DH de Bakker, FG Schellevis. Tweede Nationale Studie naar ziekten en verrichtingen in de huisartspraktijk. Klachten en aandoeningen in de bevolking en in de huisartspraktijk. Utrecht/Bilthoven: NIVEL/RIVM, 2004.
5. de Putter CE, Selles RW, Polinder S, Panneman MJ, Hovius SE, van Beeck EF. Economic impact of hand and wrist injuries: health-care costs and productivity costs in a population-based study. J Bone Joint Surg Am. 2012 May 2; 94(9).
6. Rosberg HE, Carlsson KS, Ceder-lund RI, Ramel E, Dahlin LB. Costs and outcome for serious hand and arm injuries during the first year after trauma – a prospective study. BMC Public Health 2013; 13:501.
7. Sahin F, Akca H, Akkaya N,Zincir OD, Isik A. Cost analysis and related factors in patients with traumatic hand injury. J. Hand Surg. Eur Vol 2013; 38: 673–9.
8. Schaub TA, Chung KC. Systems of provision and delivery of hand care, and its impact on the community. Injury. 2006 Nov;37(11):1066-70.
9. Taleisnik J. Clinical and technologic evaluation of ulnar wrist pain. J Hand Surg [Am] 13:801 - 802,1988.
10. Hobby JL, Tom BD, Bearcroft PW, Dixon AK. Magnetic resonance imaging of the wrist: diagnostic performance statistics. Clin Radiol. 2001 Jan; 56(1):50-7.
11. Nicholas Steel, Asmaa Abdelhamid, Tim Stokes, Helen Edwards, Robert Fleetcroft,

For peer review only

For peer review only - http://bmjopen.bmj.com/site/about/guidelines.xhtml

Amanda Howe, and Nadeem Qureshi. A review of clinical practice guidelines found that they were often based on evidence of uncertain relevance to primary care patients. J Clin Epidemiol. 2014 Nov; 67(11):1251–1257.

12. Šimundić A. Measures of Diagnostic Accuracy: Basic Definitions. EJIFCC. 2009 Jan; 19(4): 203–211.
13. Valdes K, La Stayo PJ. The value of provocative tests for the wrist and elbow: a literature review. Hand Ther. 2013 Jan-Mar; 26(1): 32-42.
14. Andersson JK, Andernord D, Karlsson J, Fridén J. Efficacy of Magnetic Resonance Imaging and Clinical Tests in Diagnostics of Wrist Ligament Injuries: A Systematic Review. Arthroscopy. 2015 Oct; 31(10):2014-20.
15. Smith TO, Drew B, Toms AP, Jerosch-Herold C, Chojnowski AJ. Diagnostic accuracy of magnetic resonance imaging and magnetic resonance arthrography for triangular fibrocartilaginous complex injury: a systematic review and meta-analysis. J Bone Joint Surg Am. 2012 May 2;94(9):824-32.
16. Moher D, Liberati A, Tetzlaff J, Altman DG; PRISMA group. Preferred reporting items for systematic reviews and meta-analyses: the PRISMA statement. BMJ. 2009; 339: b2535.
17. Gillig JD, White SD, Rachel JN. Acute Carpal Tunnel Syndrome: A Review of Current Literature.
Orthop Clin North Am. 2016 Jul;47(3):599-607.
18. Thawait GK, Subhawong TK, Thawait SK, Andreisek G, Belzberg AJ, Eng J, Carrino JA, Chhabra A. Magnetic resonance neurography of median neuropathies proximal to the carpal tunnel. Skeletal Radiol. 2012 Jun;41(6):623-32.
19. Duckworth AD, Jenkins PJ, McEachan JE. Diagnosing carpal tunnel syndrome. J Hand Surg Am. 2014 Jul;39(7):1403-7.

For peer review only

For peer review only - http://bmjopen.bmj.com/site/about/guidelines.xhtml

20. Balk EM, Chung M, Hadar N, Patel K, Yu WW, Trikalinos TA, Chang LKW. Accuracy of Data Extraction of Non-English Language Trials with Google Translate. Rockville (MD): Agency for Healthcare Research and Quality (US); 2012 Apr.

21. Worster A. and Carpenter C. Incorporation bias in studies of diagnostic tests: how to avoid being biased about bias. CJEM 2008;10(2):174-5.

22. Whiting PF, Rutjes AW, Westwood ME, Mallett S, Deeks JJ, Reitsma JB, Leeflang MM, Sterne JA, Bossuyt PM. QUADAS-2: a revised tool for the quality assessment of diagnostic accuracy studies. QUADAS-2 Group. Ann Intern Med. 2011 Oct 18; 155(8):529-36.

23. Anderson ML, Skinner JA, Felmlee JP, Berger RA, Amrami KK. Diagnostic comparison of 1.5 Tesla and 3.0 Tesla preoperative MRI of the wrist in patients with ulnar-sided wrist pain. J Hand Surg Am. 2008 Sep;33(7):1153-9.

24. Pahwa S, Srivastava DN, Sharma R, Gamanagatti S, Kotwal PP, Sharma V. Comparison of conventional MRI and MR arthrography in the evaluation wrist ligament tears: A preliminary experience. Indian J Radiol Imaging. 2014 Jul; 24(3):259-67.

25. Prosser R, Harvey L, La Stayo P, Hargreaves I, Scougall P, Herbert RD. Provocative wrist tests and MRI are of limited diagnostic value for suspected wrist ligament injuries: a cross-sectional study. J Physiother. 2011; 57(4):247-53.

26. Langner I, Fischer S, Eisenschenk A, Langner S. Cine MRI: a new approach to the diagnosis of scapholunate dissociation. Skeletal Radiol. 2015 Aug; 44(8): 1103-10.

27. Spaans AJ, Minnen Pv, Prins HJ, Korteweg MA, Schuurman AH. The value of 3.0-tesla MRI in diagnosing scapholunate ligament injury. J Wrist Surg. 2013 Feb;2(1): 69-72.

28. Greditzer HG 4th, Zeidenberg J, Kam CC, Gray RR, Clifford PD, Mintz DN, Jose J. Optimal detection of scapholunate ligament tears with MRI. Acta Radiol. 2016 Feb 9.

For peer review only - http://bmjopen.bmj.com/site/about/guidelines.xhtml

29. Al-Hiari AA. The role of wrist magnetic resonance arthrography in diagnosing triangular fibrocartilage complex tears: experience at king hussein medical center, jordan. Sultan Qaboos Univ Med J. 2013 May; 13(2):280-6.

30. Schmauss D, Pöhlmann S, Lohmeyer JA, Germann G, Bickert B, Megerle K. Clinical tests and magnetic resonance imaging have limited diagnostic value for triangular fibrocartilaginous complex lesions. Arch Orthop Trauma Surg. 2016 Jun;136(6):873-80.

31. Lee RK, Griffith JF, Ng AW, Nung RC, Yeung DK. Wrist Traction During MR Arthrography Improves Detection of Triangular Fibrocartilage Complex and Intrinsic Ligament Tears and Visibility of Articular Cartilage. AJR Am J Roentgenol. 2016 Jan;206(1):155-61.

32. Finlay K, Lee R, Friedman L. Ultrasound of intrinsic wrist ligament and triangular fibrocartilage injuries. Skeletal Radiol. 2004 Feb; 33(2): 85-90.

33. Dornberger JE, Rademacher G, Mutze S, Eisenschenk A, Stengel D. Accuracy of simple plain radiographic signs and measures to diagnose acute scapholunate ligament injuries of the wrist. Eur Radiol. 2015 Dec;25(12): 3488-98.

34. Koskinen SK, Haapamäki VV, Salo J, Lindfors NC, Kortesniemi M, Seppälä L, Mattila KT. CT arthrography of the wrist using a novel, mobile, dedicated extremity cone-beam CT (CBCT). Skeletal Radiol. 2013 May;42(5): 649-57.

35. Boer BC, Vestering M, van Raak SM, van Kooten EO, Huis In 't Veld R, Vochteloo AJH. MR arthrography is slightly more accurate than conventional MRI in detecting TFCC lesions of the wrist. Eur J Orthop Surg Traumatol. 2018 Dec;28(8):1549-1553.

36. Lee SH, Yun SJ. Point-of-care wrist ultrasonography in trauma patients with ulnar-sided pain and instability. Am J Emerg Med. 2018 May;36(5):859-864.

For peer review only - http://bmjopen.bmj.com/site/about/guidelines.xhtml

37. Suojärvi N, Haapamäki V, Lindfors N, Koskinen SK. Radiocarpal Injuries: Cone Beam Computed Tomography Arthrography, Magnetic Resonance Arthrography, and Arthroscopic Correlation among 21 Patients. Scand J Surg. 2017 Jun;106(2):173-179.

38. Mahmood A, Fountain J, Vasireddy N, Waseem M. Wrist MRI Arthrogram v Wrist Arthroscopy: What are we Finding? Open Orthop J. 2012;6:194-8.

39. Lutsky K, Levi D, Beredjiklian P. Utility of MRI for diagnosing complete tears of the collateral ligaments of the metacarpophalangeal joints of the lesser digits. Hand (N Y). 2014 Mar;9(1): 112-6.

40. Guntern D, Goncalves-Matoso V, Gray A, Picht C, Schnyder P, Theumann N. Finger A2 pulley lesions in rock climbers: detection and characterization with magnetic resonance imaging at 3 Tesla--initial results. Invest Radiol. 2007 Jun; 42(6):435-41.

41. Klauser A, Frauscher F, Bodner G, Halpern EJ, Schocke MF, Springer P, Gabl M, Judmaier W, zur Nedden D. Finger pulley injuries in extreme rock climbers: depiction with dynamic US. Radiology. 2002 Mar; 222(3):755-61.

42. Lee DH, Robbin ML, Galliott R, Graveman VA. Ultrasound evaluation of flexor tendon lacerations. J Hand Surg Am. 2000 Mar;25(2):236-41.

43. Zhang GY, Zhuang HY, Wang LX. Value of high frequency ultrasonography in diagnosis and surgical repair of traumatic finger tendon ruptures. Med Princ Pract. 2012; 21(5):472-5.

44. Mahajan M, Tolman C, Würth B, Rhemrev SJ. Clinical evaluation vs magnetic resonance imaging of the skier's thumb: A prospective cohort of 30 patients. Eur J Radiol. 2016 Oct;85(10):1750-6.

45. Shekarchi B, Mokhdanzadeh Dashti M, Shahrezaei M, Karimi E. The Accuracy of Ultrasonography in Detection of Ulnar Collateral Ligament of Thumb Injuries; a Cross-Sectional Study. Emerg (Tehran). 2018;6(1):e15.

For peer review only - http://bmjopen.bmj.com/site/about/guidelines.xhtml

46. Whiting P, Rutjes AW, Reitsma JB, Glas AS, Bossuyt PM, Kleijnen J. Sources of variation and bias in studies of diagnostic accuracy: a systematic review. Ann Intern Med. 2004 Feb 3;140(3):189-202. Review.

47. Kakar S, Breighner RE, Leng S, McCollough CH, Moran SL, Berger RA, Zhao KD. The Role of Dynamic (4D) CT in the Detection of Scapholunate Ligament Injury. J Wrist Surg. 2016 Nov;5(4):306-310.

48. Zhao K, Breighner R, Holmes III D, Leng S, McCollough C, An K. J Biomech Eng. A Technique for Quantifying Wrist Motion Using Four-Dimensional Computed Tomography: Approach and Validation. 2015 Jul;137(7).

Figure 1. Flow chart study selection

For peer review only

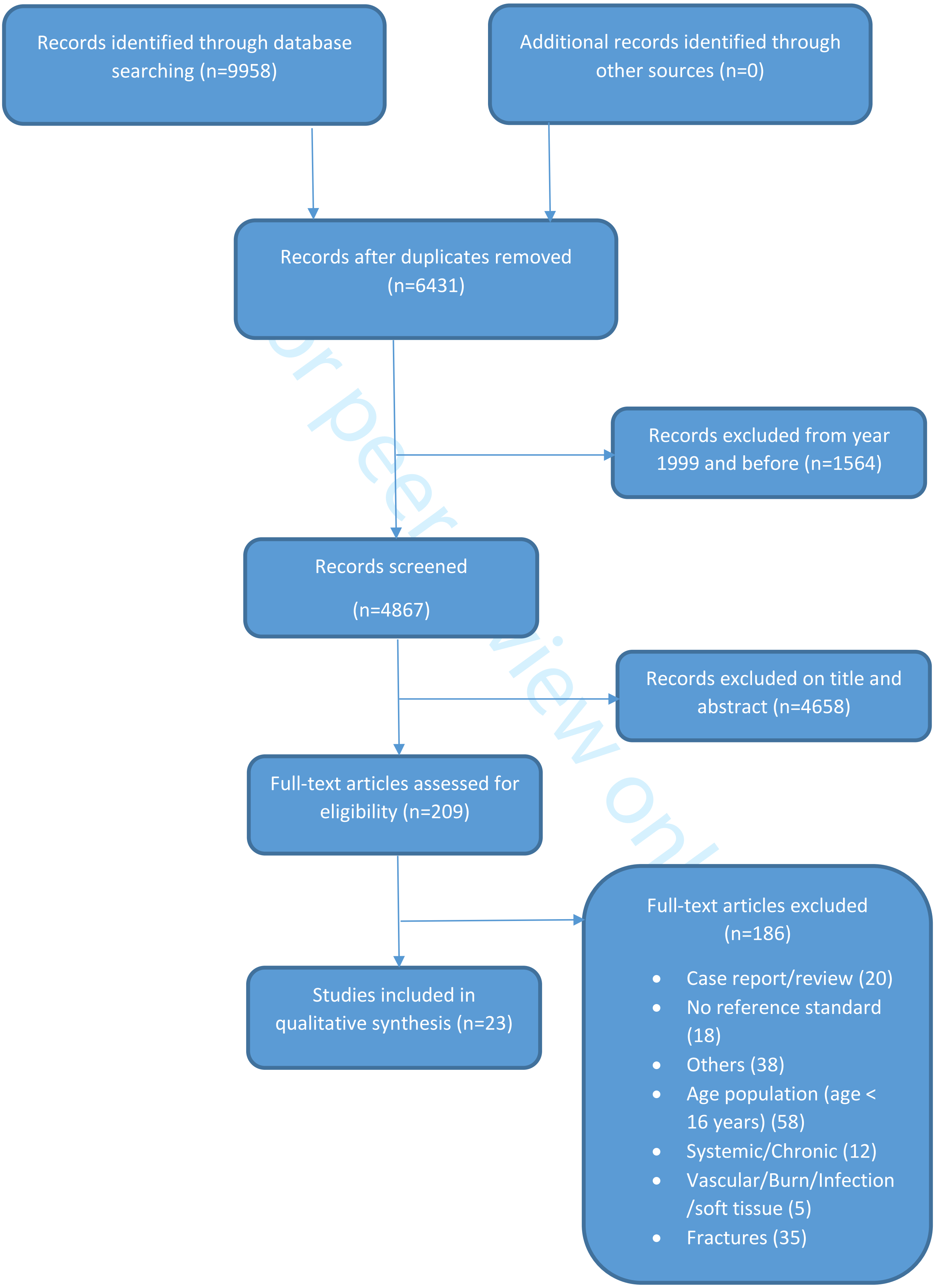
Records identified through database searching (n=9958)
Additional records identified through other sources (n=0)
Records after duplicates removed (n=6431)
Records excluded from year 1999 and before (n=1564)
Records screened
(n=4867)
Records excluded on title and abstract (n=4658)
Full-text articles assessed for eligibility (n=209)
Full-text articles excluded (n=186)
Case report/review (20)
No reference standard (18)
Others (38)
Age population (age < 16 years) (58)
Systemic/Chronic (12)
Vascular/Burn/Infection /soft tissue (5)
Fractures (35)
Studies included in qualitative synthesis (n=23)

Appendix 1

Database	Search terms
Embase	('hand injury'/exp OR 'wrist injury'/exp OR 'wrist fracture'/exp OR (('hand bone'/exp OR wrist/exp OR hand/exp OR 'wrist pain'/exp OR 'hand pain'/exp) AND ('bone injury'/exp OR fracture/de OR 'ligament injury'/exp OR 'ligament rupture'/exp)) OR (((hand OR hands OR wrist* OR finger* OR carpal* OR carpus OR phalanx* OR metacarp* OR capitate* OR hamat* OR lunat* OR pisiform* OR scaphoid* OR trapezium* OR trapezoid* OR triquetr* OR navicular* OR lunar OR semilunar* OR multangulum* OR pyramid* OR metacarpophalang* OR thumb* OR 'distal radius' OR 'distal ulna' OR 'distal radial' OR 'distal ulnar' OR scapholunate* OR lunotriquetral* OR 'triangular fibrocartilaginous' OR SLIL OR LTIL OR tfcc OR 'ulnar collateral ligament' OR 'ulnar collateral ligaments' OR ucl) NEAR/3 (injur* OR trauma* OR wound* OR lesion* OR dislocate* OR fracture* OR damage* OR tear* OR sprain* OR displace* OR rupture*))):ab,ti) AND ('diagnostic test'/de OR 'function test'/exp OR 'diagnostic error'/exp OR 'diagnostic accuracy'/exp OR 'diagnostic value'/exp OR 'differential diagnosis'/exp OR 'delayed diagnosis'/exp OR 'sensitivity and specificity'/exp OR (((diagnos* OR detect* OR differen* OR strength* OR motion*) NEAR/3 (test* OR accura* OR error* OR false OR fail* OR value* OR impact* OR effective* OR earl* OR missed OR correct* OR incorrect* OR delay* OR difficult* OR negative* OR positive* OR sensitivit* OR specificit* OR confirm* OR abilit*)) OR (diagnos* NEAR/3 differen*) OR misdiagnos* OR underdiagnos* OR undetect* OR (predict* NEAR/3 value*) OR (function* NEAR/3 test*) OR (false NEAR/3 (negative* OR positive*))):ab,ti) NOT ([Conference Abstract]/lim OR [Letter]/lim OR [Note]/lim OR [Editorial]/lim) AND [english]/lim NOT ([animals]/lim NOT [humans]/lim)

Search terms for the other databases are available upon request.

For peer review only

Appendix 2. Diagnostic Accuracy of Tests in Different Subgroups Presented in the Study of Schmauss et al.[26]

	**Se (%)**	**Sp (%)**	**Accuracy (%)**	**PPV (%)**	**NPV (%)**
Ulna carpal pain					
Ulnar fovea sign	88	9	54	56	39
Ulnar grinding test	93	12	58	58	58
MRI	88	23	58	57	61
Ulno carpal pain and prior trauma					
Ulnar fovea sign	90	8	54	55	39
Ulnar grinding test	93	12	57	57	57
MRI	93	17	59	58	65
Palmar A1 lesion					
Ulnar fovea sign	86	9	40	39	50
Ulnar grinding test	90	12	42	39	66
MRI	90	23	48	41	79
Palmer 2c Lesion					
Ulnar fovea sign	85	9	26	22	68
Ulnar grinding test	93	12	30	23	86
MRI	88	23	37	24	87

For peer review only

PRISMA 2009 Checklist

Section/topic	#	Checklist item	Reported on page #
**TITLE**			
Title	1	Identify the report as a systematic review, meta-analysis, or both.	1
**ABSTRACT**			
Structured summary	2	Provide a structured summary including, as applicable: background; objectives; data sources; study eligibility criteria, participants, and interventions; study appraisal and synthesis methods; results; limitations; conclusions and implications of key findings; systematic review registration number.	2
**INTRODUCTION**			
Rationale	3	Describe the rationale for the review in the context of what is already known.	4
Objectives	4	Provide an explicit statement of questions being addressed with reference to participants, interventions, comparisons, outcomes, and study design (PICOS).	4-5
**METHODS**			
Protocol and registration	5	Indicate if a review protocol exists, if and where it can be accessed (e.g., Web address), and, if available, provide registration information including registration number.	6
Eligibility criteria	6	Specify study characteristics (e.g., PICOS, length of follow-up) and report characteristics (e.g., years considered, language, publication status) used as criteria for eligibility, giving rationale.	7
Information sources	7	Describe all information sources (e.g., databases with dates of coverage, contact with study authors to identify additional studies) in the search and date last searched.	7
Search	8	Present full electronic search strategy for at least one database, including any limits used, such that it could be repeated.	7
Study selection	9	State the process for selecting studies (i.e., screening, eligibility, included in systematic review, and, if applicable, included in the meta-analysis).	7
Data collection process	10	Describe method of data extraction from reports (e.g., piloted forms, independently, in duplicate) and any processes for obtaining and confirming data from investigators.	8
Data items	11	List and define all variables for which data were sought (e.g., PICOS, funding sources) and any assumptions and simplifications made.	
Risk of bias in individual studies	12	Describe methods used for assessing risk of bias of individual studies (including specification of whether this was done at the study or outcome level), and how this information is to be used in any data synthesis.	8
Summary measures	13	State the principal summary measures (e.g., risk ratio, difference in means).	
Synthesis of results	14	Describe the methods of handling data and combining results of studies, if done, including measures of consistency (e.g., I^2) for each meta-analysis.	

For peer review only - http://bmjopen.bmj.com/site/about/guidelines.xhtml

PRISMA 2009 Checklist

Section/topic	#	Checklist item	Reported on page #
Risk of bias across studies	15	Specify any assessment of risk of bias that may affect the cumulative evidence (e.g., publication bias, selective reporting within studies).	
Additional analyses	16	Describe methods of additional analyses (e.g., sensitivity or subgroup analyses, meta-regression), if done, indicating which were pre-specified.	8
**RESULTS**			
Study selection	17	Give numbers of studies screened, assessed for eligibility, and included in the review, with reasons for exclusions at each stage, ideally with a flow diagram.	9
Study characteristics	18	For each study, present characteristics for which data were extracted (e.g., study size, PICOS, follow-up period) and provide the citations.	9
Risk of bias within studies	19	Present data on risk of bias of each study and, if available, any outcome level assessment (see item 12).	12
Results of individual studies	20	For all outcomes considered (benefits or harms), present, for each study: (a) simple summary data for each intervention group (b) effect estimates and confidence intervals, ideally with a forest plot.	14-19
Synthesis of results	21	Present results of each meta-analysis done, including confidence intervals and measures of consistency.	
Risk of bias across studies	22	Present results of any assessment of risk of bias across studies (see Item 15).	
Additional analysis	23	Give results of additional analyses, if done (e.g., sensitivity or subgroup analyses, meta-regression [see Item 16]).	20-21
**DISCUSSION**			
Summary of evidence	24	Summarize the main findings including the strength of evidence for each main outcome; consider their relevance to key groups (e.g., healthcare providers, users, and policy makers).	21-24
Limitations	25	Discuss limitations at study and outcome level (e.g., risk of bias), and at review-level (e.g., incomplete retrieval of identified research, reporting bias).	3
Conclusions	26	Provide a general interpretation of the results in the context of other evidence, and implications for future research.	24
**FUNDING**			
Funding	27	Describe sources of funding for the systematic review and other support (e.g., supply of data); role of funders for the systematic review.	26

From: Moher D, Liberati A, Tetzlaff J, Altman DG, The PRISMA Group (2009). Preferred Reporting Items for Systematic Reviews and Meta-Analyses: The PRISMA Statement. PLoS Med 6(7): e1000097. doi:10.1371/journal.pmed1000097

For more information, visit: **www.prisma-statement.org**.

For peer review only - http://bmjopen.bmj.com/site/about/guidelines.xhtml
